# Exploiting Self-Association to Evaluate Enantiomeric Composition by Cyclic Ion Mobility–Mass Spectrometry

**DOI:** 10.1021/acs.analchem.2c01212

**Published:** 2022-06-03

**Authors:** Dale A. Cooper-Shepherd, Hernando J. Olivos, Zhaoxiang Wu, Martin E. Palmer

**Affiliations:** † Waters Corporation, Stamford Avenue, Altrincham Road, Wilmslow SK9 4AX, United Kingdom; ‡ Waters Corporation, 34 Maple Street, Milford, Massachusetts 01757, United States

## Abstract

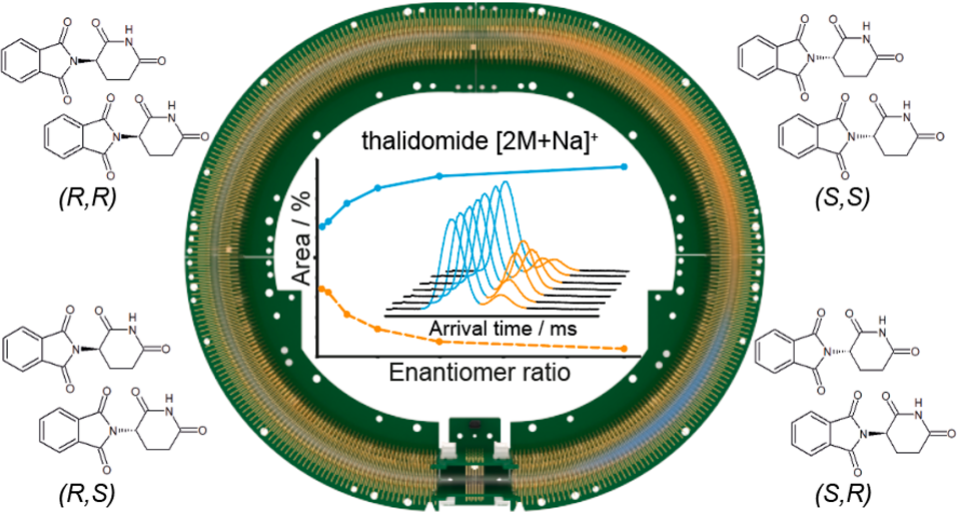

The characterization of enantiomers is an important analytical challenge in the chemical and life sciences. Thorough evaluation of the purity of chiral molecules is particularly required in the pharmaceutical industry where safety concerns are paramount. Assessment of the enantiomeric composition is still challenging and time-consuming, meaning that alternative approaches are required. In this study, we exploit the formation of dimers as diastereomeric pairs of enantiomers to affect separation by high resolution cyclic ion mobility–mass spectrometry. Using the example of (*R*/*S*)-thalidomide, we show that even though this is not an enantiomer separation, we can determine which enantiomer is in excess and obtain quantitative information on the enantiomer composition without the need for a chiral modifier. Further examples of the approach are presented, including d/l-tryptophan and (*R*/*S*)-propanolol, and demonstrate the need for mobility resolving power in excess of 400 (CCS/ΔCCS).

Mass spectrometry (MS) is invaluable in the detection and quantitation of chemical species. Over the years, the power of MS has increased with improvements in resolving power, sensitivity, and speed. A significant leap forward came when researchers in the late 1990s and early 2000s developed hybrid ion mobility–mass spectrometry systems that demonstrated great promise in the study of molecular structure.^1−5^ A further advance came in 2006 with the introduction of the first commercial ion mobility spectrometry–mass spectrometry (IMS-MS) system,^6^ which offered this powerful new tool to a broad range of scientists. Ion mobility spectrometry separates ions based on their charge, size, and shape as they are propelled through an inert buffer gas by an electric field. There are many different types of ion mobility spectrometry, including drift tube ion mobility spectrometry (DTIMS),^1−5^ trapped ion mobility spectrometry (TIMS),^7^ field-asymmetric waveform ion mobility spectrometry (FAIMS),^8^ and the focus of this Article, traveling wave ion mobility spectrometry (TWIMS).^9−11^ This latter technique has been in use for the past two decades on commercial hybrid ion mobility quadrupole time-of-flight mass spectrometers, including the recently introduced cyclic TWIMS instrument.^12^ TWIMS has also been implemented in the form of custom and commercial structures for lossless ion manipulation (SLIM)^13^ devices. Since its commercial introduction coupled to mass spectrometry, ion mobility has been used extensively in the chemical, life, and materials sciences to study molecular structure and has been particularly powerful in the separation and characterization of isomers.^14^ As they have the same chemical formulas, isomers often yield identical mass spectra, but can be differentiated by ion mobility, which measures ions by virtue of the arrangement of their atoms in three-dimensional space (i.e., configuration and conformation).

Ion mobility has been investigated extensively for the separation of optical isomers, that is, enantiomers, but this is only ever possible after introducing a chiral modifier of some sort to induce some diastereomeric character, diastereomers being readily separated by ion mobility.^15^ There has been an investigation into the separation of enantiomers by introduction of chiral dopants directly into the drift region of a DTIMS instrument.^16^ While showing promise, such work has proved difficult to reproduce.^17^ More recently, covalent derivatization prior to mobility analysis has been shown to provide an effective means to identify and quantify chiral amino acids in milk and other mixtures.^18−21^ Particularly interesting is the study of noncovalent chiral modifiers for the formation of diastereomeric complexes prior to or during electrospray ionization, which can subsequently be separated by ion mobility.^22−32^ A recent study using FAIMS has also characterized protonation-induced chirality through the formation of chiral centers at tertiary amines, leading to diastereomeric ions with distinct mobilities.^33^

Cyclic ion mobility spectrometry is a recently introduced technique that affords high mobility resolving power through the extension of the separation path length by enabling multiple passes around a cyclic TWIMS device.^12^ It has shown early promise in the characterization of isomeric species, including oligosaccharides,^34−39^ nucleosides,^40^ peptides,^41−43^ fuels,^44−46^ and native proteins.^47,48^ In particular, it is unique in its ability to perform many rounds of mobility separation punctuated by fragmentation steps, termed IMS^n^.^12,37^ Despite the ultrahigh resolution of cyclic TWIMS and any other ion mobility technique, it does not enable separation of enantiomers due to the requirement of a chiral separation environment.

In this study we investigated the separation of enantiomers by cyclic TWIMS. We observed the formation of dimers for thalidomide (thal), a model chiral pharmaceutical compound (Figure 1A). High mobility resolution analysis revealed two species for the dimers, consistent with the formation of diastereomeric pairs of enantiomers (Figure 1B). We set out to confirm the identity of these species and discovered that the observation of these dimers enabled the determination of the purity status of thal. Furthermore, the ratio of the mobility-resolved species could directly report on the amounts of each enantiomer in the mixture. By extension, therefore, we propose that this method of studying diastereomeric pairs of enantiomeric dimers could be used to rapidly estimate the enantiomeric composition of chiral species. We note that the phenomenon of self-association as a means to glean information on enantiomeric composition has been previously reported using achiral liquid chromatography^49,50^ and nuclear magnetic resonance spectroscopy (NMR).^51^ Furthermore, MS and IMS-MS have been used to study the structure and assembly of homo- and heterochiral clusters of the amino acids proline and serine.^52−61^ To our knowledge, however, this report constitutes the first instance of using ion mobility to determine enantiomeric composition directly, using only dimers where no chiral modifier is required. We investigated the generality of the dimerization phenomenon by applying the method to three other chiral molecules, tryptophan, propranolol and the covalent dimers of penicillamine disulfide and show that ultrahigh ion mobility resolution (in excess of 400 (CCS/ΔCCS) is essential for these experiments.

**Figure 1:**
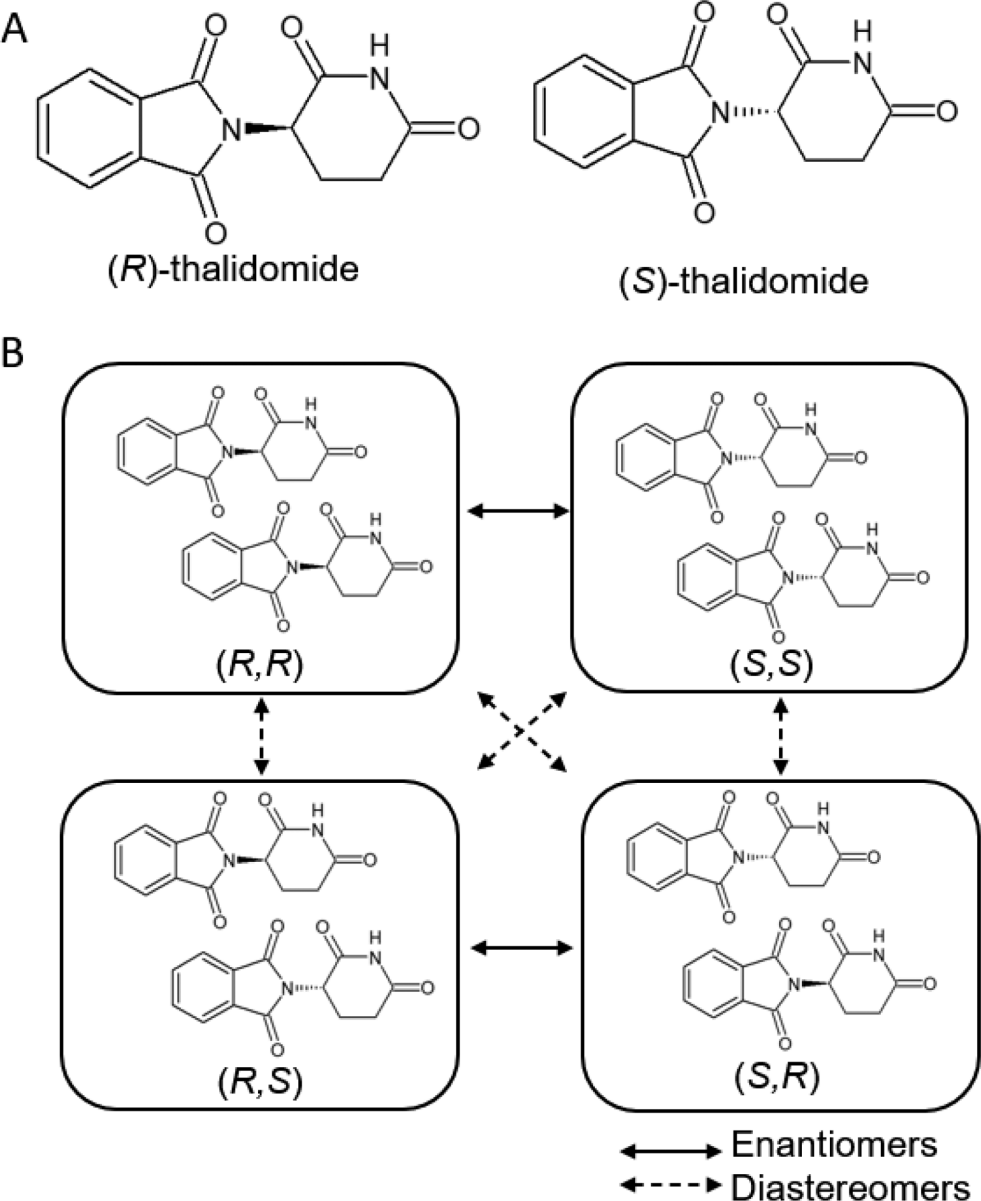
Enantiomers of thalidomide and their diastereomeric dimers. (A) The structures of (*R*)- and (*S*)-thalidomide; (B) Black boxes each contain possible dimers formed in a mixture of (*R*)- and (*S*)-thalidomide, namely (*R*,*R*), (*S*,*S*), (*R*,*S*), and (*S*,*R*). The mixed (*R*,*S*/*S*,*R*) dimers are degenerate, but are shown separately here for illustration. Solid arrows denote pairs of enantiomers, whereas dashed arrows denote pairs of diastereomers.

## Experimental Section

### Materials

All molecules studied were purchased from Merck-Sigma (Gillingham, U.K.) as follows: (*R*)-thalidomide (T151), (*S*)-thalidomide (T150), d-penicillamine (P4875), l-penicillamine (196312), l-tryptophan (T8941), d-tryptophan (T9753), (*R*)-propanolol (P0689), and (*S*)-propanolol (P8688).

### Sample Preparation

(*R*)- and (*S*)-Thalidomide solutions were prepared gravimetrically to a stock concentration of 1 mM in a solution of 50:49:1 methanol/acetonitrile/formic acid (v/v %). The stock solution was diluted to 100 μM with 50:49.9:0.1 methanol/water/formic acid (v/v %) before direct infusion into the Z-spray ion source at a flow rate of 5 μL/min. Stock solutions of d-penicillamine and l-penicillamine were prepared to a concentration of 1 mM in water and diluted to 100 μM with 50:49.9:0.1 methanol/water/formic acid (v/v %) before direct infusion. l- and d-Tryptophan and (*R*)- and (*S*)-propanolol were prepared individually to stock concentrations of 1 mM in 50:49.9:0.1 water/acetonitrile/formic acid (v/v %) and diluted to 100 μM in the same solvents prior to analysis. To simulate racemic mixtures of the compounds, equal volumes of equimolar stocks of each enantiomer were mixed before ion mobility–mass spectrometry analysis. When required, stock solutions of 1 M NaCl or 1 M LiCl were added to final concentrations of 1 mM to promote sodium and lithium adduct formation.

### Ion Mobility–Mass Spectrometry

All experiments were performed using a SELECT SERIES Cyclic IMS instrument (Waters Corporation, Wilmslow, U.K.). For high resolution multipass cyclic ion mobility analysis the traveling wave pulse height was set to 12 V, with a velocity of 375 m/s. The number of passes was varied by choosing appropriate “separate” times in the instrument control software. Pre- and postmobility voltages were minimized to preserve the dimer complexes throughout the instrument; trap and transfer collision voltages 4 and 2 V, respectively, post-trap gradient 5 V, post-trap bias 15 V, helium cell entrance 3 V, helium cell bias 22 V, array pulse height in eject 15 V, and pretransfer gradient 5 V.

For ^TW^CCS_N2_ measurements, a traveling wave height of 15 V was used with a wave velocity of 375 m/s. The instrument was CCS-calibrated with a number of ions from the CCS Majormix calibration standard (Waters part number 186008113), using a power law of the form *y* = *ax*^*b*^, where *a* and *b* are constants determined by fitting a linear regression to a natural log–log plot of reduced CCS versus arrival time.^62^

### Software and Data Analysis

Data were manually processed using Masslynx v4.2 (SCN 1016) with ^TW^CCS_N2_ values being determined using UNIFI 1.9.4 (both Waters Corp., Wilmslow, U.K.). For Gaussian fitting of arrival time distributions (ATDs), Microsoft Excel was used. For the generation of the theoretical “simplistic” scenarios, the contributions to the peak areas of the two features were calculated simply by the probabilities of dimer formation from the known mixing ratio of the two enantiomers (see Supporting Information). As discussed in the Results section, we invoke an empirical “response factor”, *F*, to adjust the simplistic peak areas to match those in the experiment.
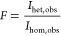
1where *I*_het_ and *I*_hom_ are the observed experimental relative peak areas of the ATDs of the heterodimer and homodimer in the racemic mixture, respectively. This response factor can then be used to determine the enantiomer ratio, E.R., for an unknown mixing ratio of enantiomers using
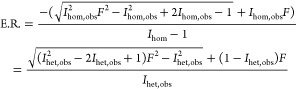
2Please refer to the Supporting Information for more details on the derivation.

## Results

### Cyclic Ion Mobility of Thalidomide Monomers and Dimers

**Figure 2:**
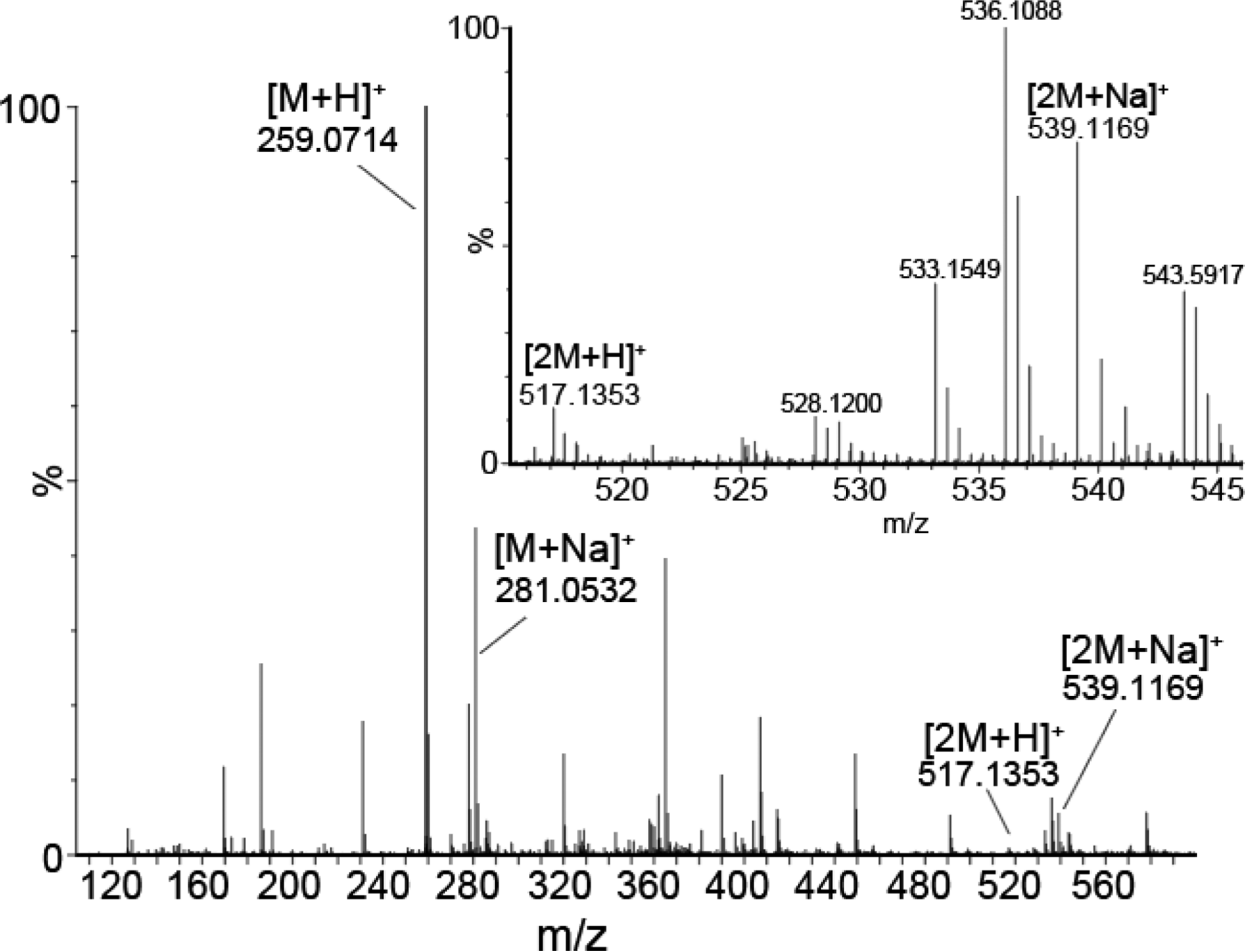
TOF-MS dimension of a single pass cyclic IMS experiment of racemic thalidomide. The major ions of interest are indicated on the spectrum, the predominant species being the [M + H]^+^ and [M + Na]^+^ at 259.07 and 281.05 *m*/*z*, respectively. Dimeric ions [2M + H]^+^ and [2M + Na]^+^ are indicated at 517.14 and 539.12 *m*/*z*, respectively. The inset shows a zoom of the region of the spectrum containing the dimer ions. A significant number of unassigned background ions are observed in the spectrum due to contamination of the flow path.

First, we acquired a single pass cyclic ion mobility (cIMS) experiment on a solution of *rac*-thalidomide to observe which species were present (Figure 2). We detected primarily the protonated form of thalidomide (thal) at 259 *m*/*z*, as well as a significant proportion of the sodiated form at 281 *m*/*z*. We also detected low levels of dimeric species at 517 *m*/*z* and 539 *m*/*z* corresponding to protonated and sodiated species, respectively. These dimers are likely formed as a result of the high solution concentrations used and the concentration effect afforded by the electrospray process.^63,64^ We note that other approaches employing noncovalent chiral modifiers use similarly high,^32^ if not higher,^30^ analyte concentrations to promote complex formation.

We performed multipass cIMS on the monomeric species at 259 and 281 *m*/*z*, the [M + H]^+^ and [M + Na]^+^, respectively. In line with expectations, no separation of species was observed, and the arrival time distributions (ATDs) obtained as well as the ^TW^CC_N2_ values were identical between *rac*-thalidomide and (*R*)- and (*S*)-thalidomide (Figure S1).

**Figure 3:**
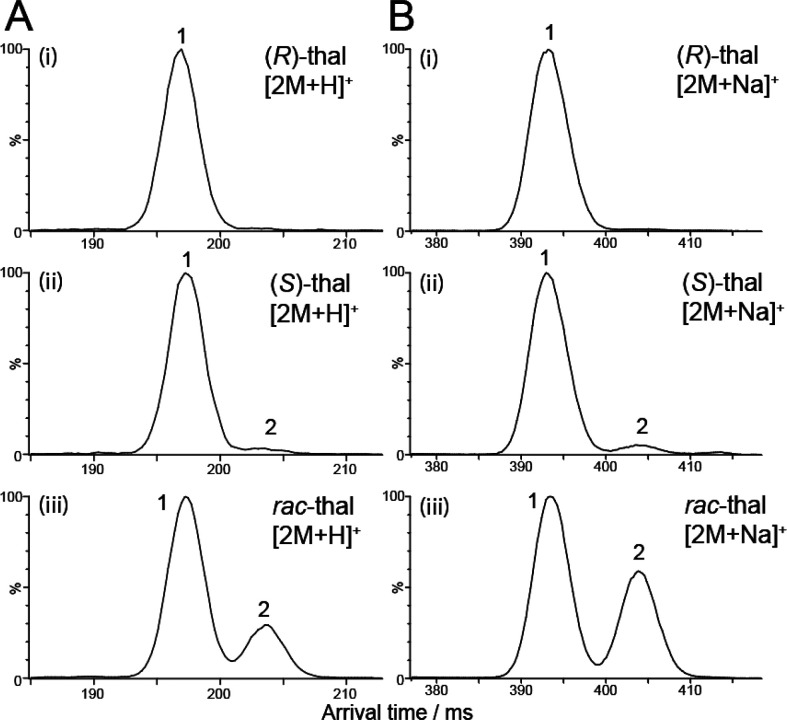
Multipass cIMS of dimeric thalidomide ions. (A) Five pass ATD of the [2M + H]^+^ ion of (i) (*R*)-thal, (ii) (*S*)-thal, and (iii) *rac*-thal. (B) Ten pass ATDs of the [2M + Na]^+^ ion of (i) (*R*)-thal, (ii) (*S*)-thal, and (iii) *rac*-thal. In both cases, *rac*-thal exhibits two features (1 and 2), albeit in different ratios. (*R*)-Thal displays overwhelmingly feature 1, whereas (*S*)-thal displays predominantly feature 1 and a small proportion of feature 2. The data are consistent with the formation of diastereomeric pairs of enantiomers.

Next, we conducted cIMS experiments on the dimeric [2M + H]^+^ and [2M + Na]^+^ ions observed at 517 *m*/*z* and 539 *m*/*z* in the *rac*-thalidomide sample, respectively. Both ion populations exhibited only a single species in their ATDs after 1 cIMS pass (Figure S2). After 5 cIMS passes (*R*_p_ ∼ 145 CCS/ΔCCS), the [2M + H]^+^ yielded two well-resolved features (Figure 3A,iii), suggesting two distinct forms of dimer. Going forward, we refer to these as features 1 and 2. The [2M + Na]^+^ ions showed similar behavior albeit after 10 cIMS passes ion (*R*_p_ ∼ 200 CCS/ΔCCS) (Figure 3B,iii), which could be baseline separated after 20 passes (Figure S2C). The ^TW^CCS_N2_ values for the dimer features were measured as 220 and 225 Å^2^ for the [2M + H]^+^ and 226 and 229 Å^2^ for [2M + Na]^+^, with differences of 2.2 and 1.3%, respectively. Interestingly, we also observed the same behavior for the lithium-adducted dimers of thal, [2M + Li]^+^, where two features were detected in the ATD of the racemic mixture, albeit with a lesser degree of separation after 10 passes (Figure S3).

The presence of two features in the dimeric ATDs implies either protomers or inherently different structures, which in this case could mean diastereomeric dimers. If the latter case is true, in the *rac*-thal sample there is the possibility of forming (*R*,*R*) and (*S*,*S*) homodimers, as well as (*R*,*S*) and (*S*,*R*) heterodimers (Figure 1B). The (*R*,*R*) and (*S*,*S*) homodimers are one enantiomer pair and the (*R*,*S*) and (*S*,*R*) heterodimers are another, with those pairs being diastereomers of each other. If the propensities of dimer formation are not drastically different for each pair, then this would give rise to two features in the ATD, one for (*R*,*R*)/(*S*,*S*) and another for (*R*,*S*)/(*S*,*R*), provided the pairs can be separated by their mobilities. Note that no separation can be achieved within each pair, as these are enantiomers.

To determine the identity of each feature, we next infused separate solutions of “pure” (*R*)- and (*S*)-thal and subjected them to the same experimental conditions. For both (*R*)-thal (Figure 3A,i and B,i) and (*S*)-thal (Figure 3A,ii and B,ii), only a single major feature was observed in each case as expected, and their arrival times were consistent only with feature 1 from *rac*-thal (Figure 3A,iii and B,iii). This supports the conclusion that feature 1 is the homodimer signal, (*R*,*R*) in the case of (*R*)-thal, (*S*,*S*) in the case of (*S*)-thal, and both (*R*,*R*) and (*S*,*S*) in *rac*-thal. Only a small indication of feature 2 was observed in the case of both protonated and sodiated (*S*)-thal (Figure 3A,ii and B,ii). The presence of feature 2 predominantly in the *rac*-thal sample leads us to conclude that this is indeed due to the (*R*,*S*)/(*S*,*R*) heterodimers that, being diastereomers of (*R*,*R*)/(*S*,*S*), are able to be separated from feature 1. Again, a similar behavior was observed for the lithiated dimers (Figure S3)

As an aside, during optimization of the experimental conditions, we noticed dissociation of dimeric ions into monomers postmobility (Figure S4). The instrument was tuned to minimize this effect, but we think it worth mentioning that if the instrument was particularly harsh, yielding only monomeric ions, it might lead to misinterpretation of the monomeric ATD as containing more than one feature, that is, as a result of retaining the mobility information of the dimeric precursors.

Returning to the dimers, a key observation in these data is the presence of feature 2 at detectable levels in both protonated and sodiated (*S*)-thal (Figures 3A,ii and B,ii). This indicates that (*S*)-thal is not pure, as there is sufficient (*R*)-thal present in solution to yield (*R*,*S*)/(*S*,*R*) heterodimers. The same is not true of the (*R*)-thal sample, which has no significant amount of the heterodimers present. This result highlights the utility of this method in providing a fast readout of the optical purity status (pure vs impure) of this chiral compound.

### Quantifying Enantiomeric Composition

**Figure 4:**
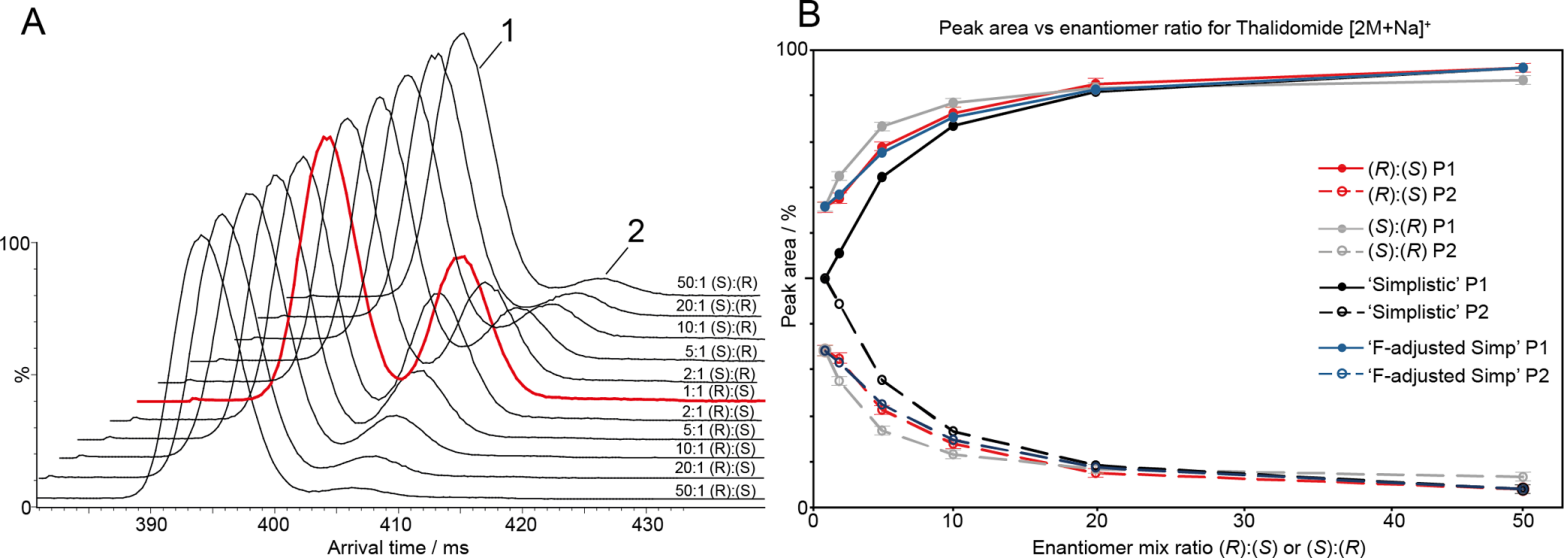
Effect of the enantiomer ratio on the observed dimer peak areas for thalidomide [2M + Na]^+^. (A) Arrival time distributions from 10 pass cIMS experiments, where the ratio of enantiomers was varied. At 50:1 (*R*)/(*S*), the relative peak area of feature 2 is low. As the amount of (*S*)-thal is increased, the relative peak area of feature 2 increases to a maximum at 1:1 (*R*)/(*S*) (red trace). As the amount of (*S*)-thal is increased further, feature two begins to decrease in intensity once more to a minimum at 50:1 (*S*)/(*R*). (B) Relative peak areas of feature 1 (P1) and feature 2 (P2) as a function of the enantiomer ratio. Red solid line, (*R*)/(*S*) P1; red dashed line, (*R*)/(*S*) P2; gray solid line, (*S*)/(*R*) P1; gray dashed line, (*S*)/(*R*) P2; black solid line and dashed line, the “simplistic” case should the dimers have the same association energy for P1 and P2, respectively; blue solid line and dashed line, the empirically *F*-adjusted “simplistic” case based on the ratios observed in the racemate P1 and P2, respectively. There is good agreement between the *F*-adjusted simplistic case and experiment.

To explore the dimerization phenomenon further we performed the multipass cIMS experiment on mixtures of (*R*)- and (*S*)-thalidomide in ratios 50:1, 20:1, 10:1, 1:1, 1:2, 1:10, 1:20, and 1:50. In this experiment we focused on the [2M + Na]^+^ ions due to their greater relative intensity in the mass spectrum and greater perceived stability. It can be seen in Figure 4A that, as the ratio of (*R*)-thal to (*S*)-thal is increased from 1:1 to 2:1, the relative intensity of feature 2 decreases. This trend is borne out as the ratio is increased further to 10:1, 20:1, and 50:1. Increasing the ratio in the opposite direction from 1:1 to 1:2 (*R*)/(*S*) exhibits similar behavior to 2:1 (*R*)/(*S*), and likewise for the rest of the mixing ratios where (*S*) is in excess. The reason for the reduced intensity of feature 2 as the enantiomer ratio deviates from 1:1 is the reduction in the relative mole fraction of the minor enantiomer available for heterodimer formation, meaning homodimers become more likely.

The relative peak areas were extracted by Gaussian fitting to the ATDs and plotted in Figure 4B. The plot reveals the increase in the relative area of feature 1 and the concomitant decrease in the area of feature 2. It was expected that the curve for the increase in (*R*)-thal (red lines) would be identical to that of (*S*)-thal (gray lines); however, significant differences are observed. The trend on increasing the proportion of (*S*)-thal from 1:1 (*S*)/(*R*) to 2:1 (*S*)/(*R*), for example, is steeper than when increasing the proportion of (*R*)-thal in the same way. This could be attributed to the fact that the (*S*)-thal sample already contains a significant amount of (*R*)-thal, as seen in Figure 3A,ii and B,ii. Despite this, the observed variation in relative peak area with enantiomer ratio reveals a second advantage of this method in that it is quantitative. The enantiomer ratio can be determined from performing this experiment on a sample containing an unknown ratio of (*R*)- and (*S*)-thalidomide. However, with this significant finding, a major caveat is revealed; the ratio of enantiomers can be determined, but the experiment does not reveal directly which enantiomer is indeed in excess. Some a priori knowledge of which enantiomer is in excess, akin to the above example of the (*S*)-thal sample, is required in order to draw conclusions on absolute enantiomer composition. However, as will be discussed below, given the appropriate pure standards the predominant enantiomer can be identified indirectly.

### Adjusting for Differences in Dimer Stability

The data in Figures 3 and 4 exhibit in all cases a relative peak area for feature 2 that is much less than feature 1, even where the enantiomer ratio is 1:1. This suggests that the heterodimers of thal are significantly less stable than the homodimers, either by virtue of the mechanism of formation during electrospray or by their susceptibility to dissociate at various stages during transfer through the instrument. For this method to be quantitative, this observation must be accounted for. Figure 4B shows a theoretical curve for the variation of relative peak areas of features 1 and 2 with enantiomer ratio given the “simplistic” theoretical case where the stability of the homo- and heterodimers are identical (black lines). This case would yield a 1:1 peak area ratio given a 1:1 mixture of (*R*)- and (*S*)-thal, which would increase according to the relative concentration of each enantiomer. In reality, the data themselves do not follow this simplistic case, so the relative stabilities of the dimers can be accounted for by a “response factor”, which we have called *F*, for the heterodimer peak based on that observed in the racemic mixture, that is, knowing the empirical peak area ratios in the 1:1 condition, we can predict the peak areas for all other enantiomer ratios (dark blue lines in Figure 4B) and use this to determine the enantiomer ratio in an unknown mixture. This “*F*-adjusted simplistic” curve in Figure 4B (dark blue lines) shows good agreement with the experimental curves, indicating that it is a good approximation of the data.

The method presented here relies on the a priori knowledge of the “response factor”, *F*, however, we note that such knowledge is not unprecedented in the study of enantiomer composition. Indeed, when determining the optical purity of a particular chiral compound using circularly polarized light, experimenters require a priori knowledge of the specific rotation of a pure enantiomer to obtain meaningful information. We suggest that *F*, in the case of the method presented here, be used in a similar way.

The above quantification method enables us to return to the supposedly “pure” (*S*)-thal ATD in Figure 3A,ii and B,ii. The quoted purity of this sample of (*S*)-thal from the manufacturer is >98%. Measuring the relative peak areas of features 1 and 2 for (*S*)-thal gives values of 96% and 4%, respectively. Applying the appropriate *F* value gives an enantiomer concentration ratio of approximately 50:1 (*S*)/(*R*), which is in excellent agreement with the quoted purity boundary of >98%.

### Applicability to Other Chiral Systems

**Figure 5:**
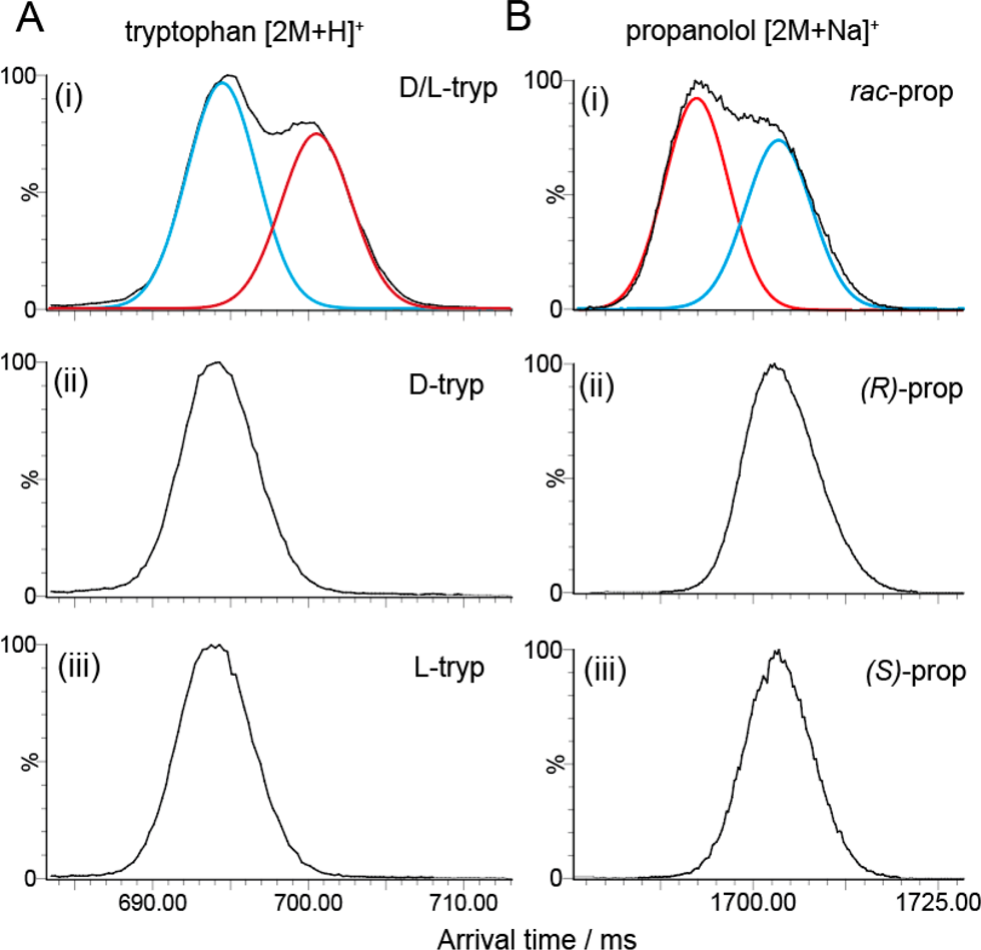
Dimerization phenomenon in other chiral systems. (A) Arrival time distributions of tryptophan [2M + H]^+^. (i) racemic d/l-tryp exhibits two features separated after 25 cIMS passes (*R* ∼ 325 CCS/ΔCCS); (ii) d-tryp exhibits a single feature as does (iii) l-tryp, indicating that the more mobile ions are the homodimers. (B) Arrival time distributions of the [2M + Na]^+^ ion of propranolol. (i) *rac*-Propanolol displays two features separated after 40 cIMS passes (*R* ∼ 411 ΔCCS/CCS), consistent with a homo- and heterodimer; (ii) (*R*)-prop exhibits a single feature, as does (iii) (*S*)-prop, indicating that the less mobile ions are the homodimers. For the ATDs of d/l-tryp and *rac*-prop, the Gaussian fits for the homodimers (blue) and heterodimers (red) are shown (A,i and B,i).

To assess the generality of the method, we next performed similar multipass cIMS experiments on the chiral compounds d/l-tryptophan (tryp) [2M + H]^+^ and (*R*)/(*S*)-propanolol (prop) [2M + Na]^+^ (Figure 5, structures shown in Figure S5). For d/l-tryptophan, 25 cIMS passes were performed yielding a mobility resolving power of approximately 325 ΔCCS/CCS. The arrival time distribution exhibited two partially resolved features, suggesting the formation of homo- and heterodimers in the same way as for thal. Performing the same experiments on isolated d- and l-tryp (Figure 5A,ii and iii) yielded identical single features for the homodimers that align with the more mobile ion population (feature 1) in the d/l-tryp ATD. This indicates that this is the homodimer and feature 2 is the heterodimer. For *rac*-prop, 40 cIMS passes were performed to gain partial separation of the dimer ions, corresponding to a mobility resolving power of 411 CCS/ΔCCS, a capability we believe is beyond any other commercial ion mobility system. Once more, performing the same experiment on isolated (*R*)- and (*S*)-prop yielded a single feature, however, this time it aligned with the less mobile population in the *rac*-prop ATD, indicating that in this case the homodimer ions have the greater CCS. While it is not surprising that the order of ion mobilities for dimeric ions observed using this method is not fixed, it does highlight that this method might be used to interrogate in greater detail the structures of the dimer ions alongside computational approaches.

Inspecting the ATDs for d/l-tryp and *rac*-prop, we also notice that the relative intensities of features 1 and 2 in the ATDs of both tryptophan and propranolol in the 1:1 condition (Figure 5A,i and B,i) indicate that their homo- and heterodimer association energies are more similar than for thal. This indicates a value of *F* closer to 1 for both of these systems.

Perturbation of the ratios of the d/l-tryp and (*R*)/(*S*)-prop enantiomers revealed the dependence of enantiomer composition on the relative areas of the two features (Figure S6), indicating that, in the same way as for thal, these ratios can be used to determine enantiomer composition, given the appropriate prior information. For both tryptophan and propranolol, Gaussian peak fitting was performed to determine the relative peak areas. For each, the empirical *F* value was determined from the 1:1 condition and applied to the other ratios, and excellent agreement was observed between the corresponding “*F*-adjusted simplistic” curves and the experimental data (Figure S7).

A further example of the dimerization phenomenon was found in penicillamine (Figure S5) that, rather than forming noncovalent electrospray-mediated dimers, forms spontaneous disulfide-linked dimers. Performing a 10 pass cIMS experiment on the d/l-penicillamine mixture yielded two features, with only one in the isolated d- and l-forms (Figure S8). While this provides an additional example of separating pairs of diastereomeric pairs of enantiomeric dimers, it may be more difficult to probe relative enantiomer ratios in this case due to the predominance of irreversible covalent dimer formation.

### Determining Enantiomer Identity

**Figure 6:**
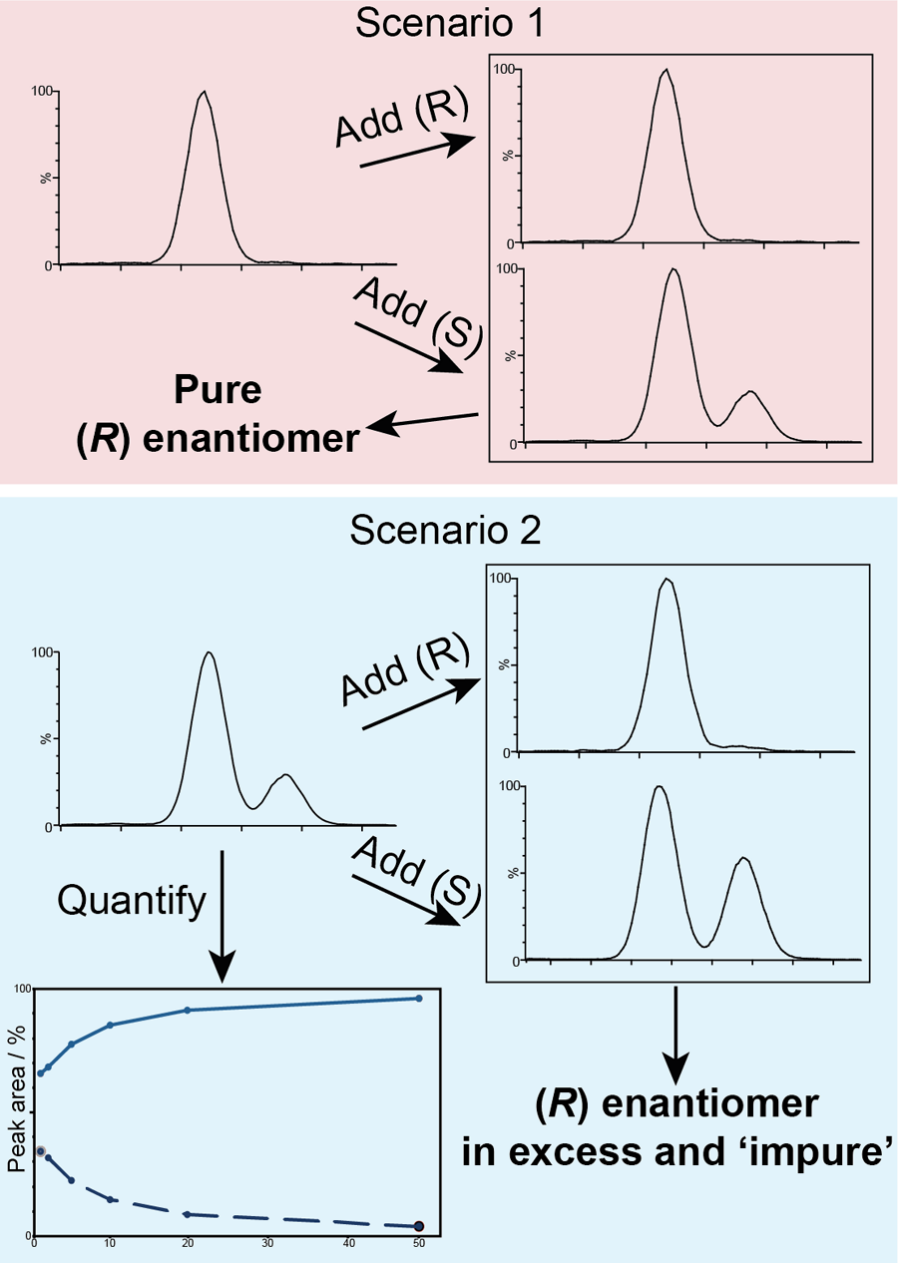
Summary of the method. Scenario 1 (top), where the experimenter is faced with a single feature in the ATD. If, for example, some pure (*R*) enantiomer is added, the ATD appearance will not change and would identify the starting substance as (*R*). If (*S*) is added, however, a second heterodimer feature will appear, supporting the conclusion that the starting substance is (*R*). The converse is true of the pure (*S*) enantiomer. Scenario 2 (bottom), where a bimodal distribution is observed. Adding (*R*) will increase the relative peak area of feature 1 and decrease feature 2. Adding (*S*) will increase the relative area of feature 2. Both these observations would identify (*R*) as being in excess. The original ATD can be used to quantify the enantiomer composition.

It is worth discussing that this method may be used to determine which enantiomer is present in excess in a sample, albeit indirectly, by performing a titration, given that sample is nonracemic. Imagine a pure enantiomer of unknown identity, where the experimental homodimer ion mobility trace exhibits a single peak, as in Figure 3A and B,i,ii. Should the user have a pure standard available of either enantiomer, two mixtures, one of the unknown with the (*R*) enantiomer and one with the (*S*) enantiomer, can be prepared. If the unknown is actually the (*R*) enantiomer, after mixing with the (*R*) enantiomer the ion mobility trace will not change (Figure 6, top). In the case of mixing with the (*S*) enantiomer the ion mobility trace will exhibit a second peak similar to feature 2 in the above data (Figure 3A,ii and B,ii). The same is true if the unknown sample is not completely pure. If more (*S*) is added, the intensity of the heterodimer peak will increase, if more (*R*) is added the intensity of the homodimer peak will increase (Figure 6, bottom). Hence, the approach has a third key utility, determining which enantiomer is in excess. It should be noted, however, that when probing a near racemic mixture with this titration experiment care must be taken not to “overshoot” the racemate as this could lead to misleading results.

## Conclusions

The characterization of chiral systems is a significant challenge in analytical chemistry, and faster and more informative techniques are required to serve the chemical and life science fields. Separation of enantiomers by imposing a diastereomeric character either covalently or noncovalently requires specific and nonuniversal derivatizing agents or cofactors as chiral modifiers. We have presented here a simple and rapid approach to the determination of the enantiomeric composition of chiral compounds without the need for any chiral modifier. We have shown that by forming diastereomeric pairs of enantiomeric dimers and separating them by high resolution cyclic ion mobility–mass spectrometry, we can determine the purity status and the enantiomer ratio and, given the appropriate available pure materials, we can determine the identity of the major and minor components in an enantiomer mixture. We believe this approach will be highly valuable in particular in the pharmaceutical industry where the in-depth knowledge of drug substances is both a safety and an intellectual property concern.

## References

[ref1] Clemmer D. E.; Hudgins R. R.; Jarrold M. F. Naked Protein Conformations: Cytochrome c in the Gas Phase. J. Am. Chem. Soc. 1995, 117 (40), 10141–10142. 10.1021/ja00145a037.

[ref2] Wu C.; Siems W. F.; Asbury G. R.; Hill H. H. Electrospray Ionization High-Resolution Ion Mobility Spectrometry–Mass Spectrometry. Anal. Chem. 1998, 70 (23), 4929–4938. 10.1021/ac980414z. 21644676

[ref3] Hoaglund C. S.; Valentine S. J.; Sporleder C. R.; Reilly J. P.; Clemmer D. E. Three-Dimensional Ion Mobility/TOFMS Analysis of Electrosprayed Biomolecules. Anal. Chem. 1998, 70 (11), 2236–2242. 10.1021/ac980059c. 9624897

[ref4] Wyttenbach T.; Kemper P. R.; Bowers M. T. Design of a New Electrospray Ion Mobility Mass Spectrometer. Int. J. Mass Spectrom. 2001, 212 (1), 13–23. 10.1016/S1387-3806(01)00517-6.

[ref5] Valentine S. J.; Koeniger S. L.; Clemmer D. E. A Split-Field Drift Tube for Separation and Efficient Fragmentation of Biomolecular Ions. Anal. Chem. 2003, 75 (22), 6202–6208. 10.1021/ac030111r. 14616002

[ref6] Pringle S. D.; Giles K.; Wildgoose J. L.; Williams J. P.; Slade S. E.; Thalassinos K.; Bateman R. H.; Bowers M. T.; Scrivens J. H. An Investigation of the Mobility Separation of Some Peptide and Protein Ions Using a New Hybrid Quadrupole/Travelling Wave IMS/Oa-ToF Instrument. Int. J. Mass Spectrom. 2007, 261 (1), 1–12. 10.1016/j.ijms.2006.07.021.

[ref7] Michelmann K.; Silveira J. A.; Ridgeway M. E.; Park M. A. Fundamentals of Trapped Ion Mobility Spectrometry. J. Am. Soc. Mass Spectrom. 2015, 26 (1), 14–24. 10.1007/s13361-014-0999-4. 25331153

[ref8] Guevremont R. High-Field Asymmetric Waveform Ion Mobility Spectrometry: A New Tool for Mass Spectrometry. Journal of Chromatography A 2004, 1058 (1), 3–19. 10.1016/S0021-9673(04)01478-5. 15595648

[ref9] Giles K.; Pringle S. D.; Worthington K. R.; Little D.; Wildgoose J. L.; Bateman R. H. Applications of a Travelling Wave-Based Radio-Frequency-Only Stacked Ring Ion Guide. Rapid Commun. Mass Spectrom. 2004, 18 (20), 2401–2414. 10.1002/rcm.1641. 15386629

[ref10] Thalassinos K.; Slade S. E.; Jennings K. R.; Scrivens J. H.; Giles K.; Wildgoose J.; Hoyes J.; Bateman R. H.; Bowers M. T. Ion Mobility Mass Spectrometry of Proteins in a Modified Commercial Mass Spectrometer. Int. J. Mass Spectrom. 2004, 236 (1–3), 55–63. 10.1016/j.ijms.2004.05.008.

[ref11] Shvartsburg A. A.; Smith R. D. Fundamentals of Traveling Wave Ion Mobility Spectrometry. Anal. Chem. 2008, 80 (24), 9689–9699. 10.1021/ac8016295. 18986171 PMC2761765

[ref12] Giles K.; Ujma J.; Wildgoose J.; Pringle S.; Richardson K.; Langridge D.; Green M. A Cyclic Ion Mobility-Mass Spectrometry System. Anal. Chem. 2019, 91 (13), 8564–8573. 10.1021/acs.analchem.9b01838. 31141659

[ref13] Ibrahim Y. M.; Hamid A. M.; Deng L.; Garimella S. V. B.; Webb I. K.; Baker E. S.; Smith R. D. New Frontiers for Mass Spectrometry Based upon Structures for Lossless Ion Manipulations. Analyst 2017, 142 (7), 1010–1021. 10.1039/C7AN00031F. 28262893 PMC5431593

[ref14] Wu Q.; Wang J.-Y.; Han D.-Q.; Yao Z.-P. Recent Advances in Differentiation of Isomers by Ion Mobility Mass Spectrometry. TrAC Trends in Analytical Chemistry 2020, 124, 115801 10.1016/j.trac.2019.115801.

[ref15] Zhang J. D.; Mohibul Kabir K. M.; Donald W. A. Ion-Mobility Mass Spectrometry for Chiral Analysis of Small Molecules. Comprehensive Analytical Chemistry; Elsevier, 2019; Vol. 83, pp 51–81, 10.1016/bs.coac.2018.08.009.

[ref16] Dwivedi P.; Wu C.; Matz L. M.; Clowers B. H.; Siems W. F.; Hill H. H. Gas-Phase Chiral Separations by Ion Mobility Spectrometry. Anal. Chem. 2006, 78 (24), 8200–8206. 10.1021/ac0608772. 17165808 PMC3633475

[ref17] Hill H. H. Comment on “Gas-Phase Chiral Separations by Ion Mobility Spectrometry. Anal. Chem. 2022, 94, 3020 10.1021/acs.analchem.1c04903. 35099938

[ref18] Tian H.; Zheng N.; Li S.; Zhang Y.; Zhao S.; Wen F.; Wang J. Characterization of Chiral Amino Acids from Different Milk Origins Using Ultra-Performance Liquid Chromatography Coupled to Ion-Mobility Mass Spectrometry. Sci. Rep 2017, 7 (1), 46289 10.1038/srep46289. 28393862 PMC5385494

[ref19] Pérez-Míguez R.; Bruyneel B.; Castro-Puyana M.; Marina M. L.; Somsen G. W.; Domínguez-Vega E. Chiral Discrimination of DL-Amino Acids by Trapped Ion Mobility Spectrometry after Derivatization with (+)-1-(9-Fluorenyl)Ethyl Chloroformate. Anal. Chem. 2019, 91 (5), 3277–3285. 10.1021/acs.analchem.8b03661. 30682252 PMC6404107

[ref20] Campbell J. L.; Kafle A.; Bowman Z.; Blanc J. C. Y. L.; Liu C.; Hopkins W. S. Separating Chiral Isomers of Amphetamine and Methamphetamine Using Chemical Derivatization and Differential Mobility Spectrometry. Analytical Science Advances 2020, 1 (4), 233–244. 10.1002/ansa.202000066. PMC10989161 38716384

[ref21] Will J. M.; Behrens A.; Macke M.; Quarles C. D.; Karst U. Automated Chiral Analysis of Amino Acids Based on Chiral Derivatization and Trapped Ion Mobility–Mass Spectrometry. Anal. Chem. 2021, 93 (2), 878–885. 10.1021/acs.analchem.0c03481. 33337156

[ref22] Mie A.; Jörntén-Karlsson M.; Axelsson B.-O.; Ray A.; Reimann C. T. Enantiomer Separation of Amino Acids by Complexation with Chiral Reference Compounds and High-Field Asymmetric Waveform Ion Mobility Spectrometry: Preliminary Results and Possible Limitations. Anal. Chem. 2007, 79 (7), 2850–2858. 10.1021/ac0618627. 17326611

[ref23] Mie A.; Ray A.; Axelsson B.-O.; Jörntén-Karlsson M.; Reimann C. T. Terbutaline Enantiomer Separation and Quantification by Complexation and Field Asymmetric Ion Mobility Spectrometry–Tandem Mass Spectrometry. Anal. Chem. 2008, 80 (11), 4133–4140. 10.1021/ac702262k. 18447322

[ref24] Domalain V.; Hubert-Roux M.; Tognetti V.; Joubert L.; Lange C. M.; Rouden J.; Afonso C. Enantiomeric Differentiation of Aromatic Amino Acids Using Traveling Wave Ion Mobility-Mass Spectrometry. Chem. Sci. 2014, 5 (8), 3234–3239. 10.1039/C4SC00443D.

[ref25] Yu X.; Yao Z.-P. Chiral Differentiation of Amino Acids through Binuclear Copper Bound Tetramers by Ion Mobility Mass Spectrometry. Anal. Chim. Acta 2017, 981, 62–70. 10.1016/j.aca.2017.05.026. 28693730

[ref26] Zhang J. D.; Kabir K. M. M.; Donald W. A. Metal-Ion Free Chiral Analysis of Amino Acids as Small as Proline Using High-Definition Differential Ion Mobility Mass Spectrometry. Anal. Chim. Acta 2018, 1036, 172–178. 10.1016/j.aca.2018.06.026. 30253829

[ref27] Nagy G.; Chouinard C. D.; Attah I. K.; Webb I. K.; Garimella S. V. B.; Ibrahim Y. M.; Baker E. S.; Smith R. D. Distinguishing Enantiomeric Amino Acids with Chiral Cyclodextrin Adducts and Structures for Lossless Ion Manipulations. ELECTROPHORESIS 2018, 39 (24), 3148–3155. 10.1002/elps.201800294. 30168603 PMC6294673

[ref28] Gu L.; Yang S.; Wu F.; Xu F.; Yu S.; Zhou M.; Chu Y.; Ding C. Enantio-separation of Pregabalin by Ternary Complexation Using Trapped Ion Mobility Spectrometry. Rapid Commun. Mass Spectrom. 2021, 35 (8), na 10.1002/rcm.9052. 33470461

[ref29] Xie C.; Gu L.; Wu Q.; Li L.; Wang C.; Yu J.; Tang K. Effective Chiral Discrimination of Amino Acids through Oligosaccharide Incorporation by Trapped Ion Mobility Spectrometry. Anal. Chem. 2021, 93 (2), 859–867. 10.1021/acs.analchem.0c03461. 33226780

[ref30] Yang S.; Wu F.; Yu F.; Gu L.; Wang H.; Liu Y.; Chu Y.; Wang F.; Fang X.; Ding C.-F. Distinction of Chiral Penicillamine Using Metal-Ion Coupled Cyclodextrin Complex as Chiral Selector by Trapped Ion Mobility-Mass Spectrometry and a Structure Investigation of the Complexes. Anal. Chim. Acta 2021, 1184, 339017 10.1016/j.aca.2021.339017. 34625257

[ref31] Li Y.; Zhou B.; Wang K.; Zhang J.; Sun W.; Zhang L.; Guo Y. Powerful Steroid-Based Chiral Selector for High-Throughput Enantiomeric Separation of α-Amino Acids Utilizing Ion Mobility–Mass Spectrometry. Anal. Chem. 2021, 93 (40), 13589–13596. 10.1021/acs.analchem.1c02691. 34597017

[ref32] Zhang J. D.; Mohibul Kabir K. M.; Lee H. E.; Donald W. A. Chiral Recognition of Amino Acid Enantiomers Using High-Definition Differential Ion Mobility Mass Spectrometry. Int. J. Mass Spectrom. 2018, 428, 1–7. 10.1016/j.ijms.2018.02.003.

[ref33] Ieritano C.; Le Blanc J. C. Y.; Schneider B. B.; Bissonnette J. R.; Haack A.; Hopkins W. S. Protonation-Induced Chirality Drives Separation by Differential Ion Mobility Spectrometry. Angew. Chem., Int. Ed. Engl. 2022, 61, e202116794 10.1002/anie.202116794. 34963024

[ref34] Ujma J.; Ropartz D.; Giles K.; Richardson K.; Langridge D.; Wildgoose J.; Green M.; Pringle S. Cyclic Ion Mobility Mass Spectrometry Distinguishes Anomers and Open-Ring Forms of Pentasaccharides. J. Am. Soc. Mass Spectrom. 2019, 30 (6), 1028–1037. 10.1007/s13361-019-02168-9. 30977045 PMC6517361

[ref35] Ropartz D.; Fanuel M.; Ujma J.; Palmer M.; Giles K.; Rogniaux H. Structure Determination of Large Isomeric Oligosaccharides of Natural Origin through Multipass and Multistage Cyclic Traveling-Wave Ion Mobility Mass Spectrometry. Anal. Chem. 2019, 91 (18), 12030–12037. 10.1021/acs.analchem.9b03036. 31449397

[ref36] Ollivier S.; Tarquis L.; Fanuel M.; Li A.; Durand J.; Laville E.; Potocki-Veronese G.; Ropartz D.; Rogniaux H. Anomeric Retention of Carbohydrates in Multistage Cyclic Ion Mobility (IMS ^*n*^): De Novo Structural Elucidation of Enzymatically Produced Mannosides. Anal. Chem. 2021, 93 (15), 6254–6261. 10.1021/acs.analchem.1c00673. 33829764

[ref37] Ollivier S.; Fanuel M.; Rogniaux H.; Ropartz D. Molecular Networking of High-Resolution Tandem Ion Mobility Spectra: A Structurally Relevant Way of Organizing Data in Glycomics?. Anal. Chem. 2021, 93 (31), 10871–10878. 10.1021/acs.analchem.1c01244. 34324299

[ref38] Peterson T. L.; Nagy G. Toward Sequencing the Human Milk Glycome: High-Resolution Cyclic Ion Mobility Separations of Core Human Milk Oligosaccharide Building Blocks. Anal. Chem. 2021, 93 (27), 9397–9407. 10.1021/acs.analchem.1c00942. 34185494

[ref39] Williamson D. L.; Bergman A. E.; Nagy G. Investigating the Structure of α/β Carbohydrate Linkage Isomers as a Function of Group I Metal Adduction and Degree of Polymerization as Revealed by Cyclic Ion Mobility Separations. J. Am. Soc. Mass Spectrom. 2021, 32 (10), 2573–2582. 10.1021/jasms.1c00207. 34464117

[ref40] Kenderdine T.; Nemati R.; Baker A.; Palmer M.; Ujma J.; FitzGibbon M.; Deng L.; Royzen M.; Langridge J.; Fabris D. High-Resolution Ion Mobility Spectrometry-Mass Spectrometry of Isomeric/Isobaric Ribonucleotide Variants. Journal of Mass Spectrometry 2020, 55 (2), e4465 10.1002/jms.4465. 31697854 PMC8363168

[ref41] Liu Y.; Liu Y.; Nytka M.; Huang S. R.; Lemr K.; Tureček F. Probing D- and l-Adrenaline Binding to B2-Adrenoreceptor Peptide Motifs by Gas-Phase Photodissociation Cross-Linking and Ion Mobility Mass Spectrometry. J. Am. Soc. Mass Spectrom. 2021, 32 (4), 1041–1052. 10.1021/jasms.1c00019. 33655750

[ref42] Deslignière E.; Botzanowski T.; Diemer H.; Cooper-Shepherd D. A.; Wagner-Rousset E.; Colas O.; Béchade G.; Giles K.; Hernandez-Alba O.; Beck A.; Cianférani S. High-Resolution IMS–MS to Assign Additional Disulfide Bridge Pairing in Complementarity-Determining Regions of an IgG4Monoclonal Antibody. J. Am. Soc. Mass Spectrom. 2021, 32 (10), 2505–2512. 10.1021/jasms.1c00151. 34437803

[ref43] Tomczyk N.; Giles K.; Richardson K.; Ujma J.; Palmer M.; Nielsen P. K.; Haselmann K. F. Mapping Isomeric Peptides Derived from Biopharmaceuticals Using High-Resolution Ion Mobility Mass Spectrometry. Anal. Chem. 2021, 93 (49), 16379–16384. 10.1021/acs.analchem.1c02834. 34842410

[ref44] Cho E.; Riches E.; Palmer M.; Giles K.; Ujma J.; Kim S. Isolation of Crude Oil Peaks Differing by m/z ∼ 0.1 via Tandem Mass Spectrometry Using a Cyclic Ion Mobility-Mass Spectrometer. Anal. Chem. 2019, 91 (22), 14268–14274. 10.1021/acs.analchem.9b02255. 31613096

[ref45] Cho E.; Cho Y.; Rakhmat S.; Kim Y. H.; Kim S. Molecular-Level Structural Analysis of Hydrotreated and Untreated Atmospheric Residue Oils via Atmospheric Pressure Photoionization Cyclic Ion Mobility Mass Spectrometry and Ultrahigh-Resolution Mass Spectrometry. Energy Fuels 2021, 35 (22), 18163–18169. 10.1021/acs.energyfuels.1c02369.

[ref46] Rüger C. P.; Le Maître J.; Maillard J.; Riches E.; Palmer M.; Afonso C.; Giusti P. Exploring Complex Mixtures by Cyclic Ion Mobility High-Resolution Mass Spectrometry: Application Toward Petroleum. Anal. Chem. 2021, 93 (14), 5872–5881. 10.1021/acs.analchem.1c00222. 33784070

[ref47] Eldrid C.; Ujma J.; Kalfas S.; Tomczyk N.; Giles K.; Morris M.; Thalassinos K. Gas Phase Stability of Protein Ions in a Cyclic Ion Mobility Spectrometry Traveling Wave Device. Anal. Chem. 2019, 91 (12), 7554–7561. 10.1021/acs.analchem.8b05641. 31117399 PMC7006968

[ref48] Eldrid C.; Ben-Younis A.; Ujma J.; Britt H.; Cragnolini T.; Kalfas S.; Cooper-Shepherd D.; Tomczyk N.; Giles K.; Morris M.; Akter R.; Raleigh D.; Thalassinos K. Cyclic Ion Mobility–Collision Activation Experiments Elucidate Protein Behavior in the Gas Phase. J. Am. Soc. Mass Spectrom. 2021, 32 (6), 1545–1552. 10.1021/jasms.1c00018. 34006100 PMC8172447

[ref49] Wzorek A.; Sato A.; Drabowicz J.; Soloshonok V. A. Self-Disproportionation of Enantiomers (SDE) of Chiral Nonracemic Amides via Achiral Chromatography. Isr. J. Chem. 2016, 56 (11–12), 977–989. 10.1002/ijch.201600077.

[ref50] Dobashi A.; Motoyama Y.; Kinoshita K.; Hara S.; Fukasaku N. Self-Induced Chiral Recognition in the Association of Enantiomeric Mixtures on Silica Gel Chromatography. Anal. Chem. 1987, 59 (17), 2209–2211. 10.1021/ac00144a043.

[ref51] Szakács Z.; Sánta Z.; Lomoschitz A.; Szántay C. Self-Induced Recognition of Enantiomers (SIRE) and Its Application in Chiral NMR Analysis. TrAC Trends in Analytical Chemistry 2018, 109, 180–197. 10.1016/j.trac.2018.07.020.

[ref52] Julian R. R.; Myung S.; Clemmer D. E. Spontaneous Anti-Resolution in Heterochiral Clusters of Serine. J. Am. Chem. Soc. 2004, 126 (13), 4110–4111. 10.1021/ja031516w. 15053592

[ref53] Myung S.; Julian R. R.; Nanita S. C.; Cooks R. G.; Clemmer D. E. Formation of Nanometer-Scale Serine Clusters by Sonic Spray. J. Phys. Chem. B 2004, 108 (19), 6105–6111. 10.1021/jp037482w.

[ref54] Nanita S. C.; Takats Z.; Cooks R. G.; Myung S.; Clemmer D. E. Chiral Enrichment of Serine via Formation, Dissociation, and Soft-Landing of Octameric Cluster Ions. J. Am. Soc. Mass Spectrom. 2004, 15 (9), 1360–1365. 10.1016/j.jasms.2004.06.010. 15337517

[ref55] Julian R. R.; Myung S.; Clemmer D. E. Do Homochiral Aggregates Have an Entropic Advantage?. J. Phys. Chem. B 2005, 109 (1), 440–444. 10.1021/jp046478x. 16851034

[ref56] Myung S.; Fioroni M.; Julian R. R.; Koeniger S. L.; Baik M.-H.; Clemmer D. E. Chirally Directed Formation of Nanometer-Scale Proline Clusters. J. Am. Chem. Soc. 2006, 128 (33), 10833–10839. 10.1021/ja0622711. 16910678

[ref57] Myung S.; Lorton K. P.; Merenbloom S. I.; Fioroni M.; Koeniger S. L.; Julian R. R.; Baik M.-H.; Clemmer D. E. Evidence for Spontaneous Resolution of Icosahedral Proline. J. Am. Chem. Soc. 2006, 128 (50), 15988–15989. 10.1021/ja066278u. 17165723

[ref58] Atlasevich N.; Holliday A. E.; Valentine S. J.; Clemmer D. E. Collisional Activation of [14Pro+2H]2+ Clusters: Chiral Dependence of Evaporation and Fission Processes. J. Phys. Chem. B 2012, 116 (26), 7644–7651. 10.1021/jp303778w. 22668003 PMC3503484

[ref59] Atlasevich N.; Holliday A. E.; Valentine S. J.; Clemmer D. E. Chirality and Packing in Small Proline Clusters. J. Phys. Chem. B 2012, 116 (37), 11442–11446. 10.1021/jp3069915. 22916685

[ref60] Holliday A. E.; Atlasevich N.; Myung S.; Plasencia M. D.; Valentine S. J.; Clemmer D. E. Oscillations of Chiral Preference in Proline Clusters. J. Phys. Chem. A 2013, 117 (6), 1035–1041. 10.1021/jp302677n. 22668126

[ref61] Jacobs A. D.; Jovan Jose K. V.; Horness R.; Raghavachari K.; Thielges M. C.; Clemmer D. E. Cooperative Formation of Icosahedral Proline Clusters from Dimers. J. Am. Soc. Mass Spectrom. 2018, 29 (1), 95–102. 10.1007/s13361-017-1833-6. 29127569 PMC6884317

[ref62] Smith D. P.; Knapman T. W.; Campuzano I.; Malham R. W.; Berryman J. T.; Radford S. E.; Ashcroft A. E. Deciphering Drift Time Measurements from Travelling Wave Ion Mobility Spectrometry-Mass Spectrometry Studies. Eur. J. Mass Spectrom (Chichester) 2009, 15 (2), 113–130. 10.1255/ejms.947. 19423898

[ref63] Wang H.; Agnes G. R. Evaluation of Electrospray Mass Spectrometry as a Technique for Quantitative Analysis of Kinetically Labile Solution Species. Anal. Chem. 1999, 71 (17), 3785–3792. 10.1021/ac9813742. 21662883

[ref64] Wang H.; Agnes G. R. Kinetically Labile Equilibrium Shifts Induced by the Electrospray Process. Anal. Chem. 1999, 71 (19), 4166–4172. 10.1021/ac981375u. 21662847

