# Supplementary material for: Exploiting Self-Association to Evaluate Enantiomeric Composition by Cyclic Ion Mobility–Mass Spectrometry
Source: Anal Chem. 2022 Jun 3;94(23):8441–8. doi: 10.1021/acs.analchem.2c01212 (PMC9201813; doi:10.1021/acs.analchem.2c01212)
Supplement: Supplementary file 1 — ac2c01212_si_001.pdf [file ac2c01212_si_001.pdf]

Supplementary Information

Exploiting self-association to evaluate enantiomeric composition by cyclic ion mobility-mass spectrometry

Dale A. Cooper-Shepherd[1]*, Hernando J. Olivos[2] and Zhaoxiang Wu[2] , Martin E. Palmer[1]

1. Waters Corporation, Stamford Avenue, Altrincham Road, Wilmslow, SK9 4AX, U.K.
2. Waters Corporation, 34 Maple Street, Milford, MA, 01757, U.S.A.

Table of Contents

Figure S1. Multipass cyclic IMS of monomeric thal. S2

Figure S2. Single and multipass cyclic IMS of dimerc thal S3

Figure S3. Ten pass cyclic ion mobility experiments of lithium adducts of thalidomide dimers at 523 m/z S4

Figure S4. The pitfalls of harsh tuning on the dimer separation experiments S5

Figure S5. Structures of the chiral compounds studied in this work S6

Figure S6. Arrival time distributions of the [2M+H]+ of D/L-tryp (A) and [2M+Na]+ of (R)/(S)-prop (B) at ratios of 10:1, 2:1, 1:1, 1:2 and 1:10 S7

Figure S7. Plots of relative peak areas vs enantiomer ratios for D/L-tryp and (R)/(S)-prop S8

Figure S8. Ten pass cIMS of covalent dimers of D/L-penicillamine S9

Supplementary Methods – Calculation of theoretical relative peak areas for homo- and heterodimeric species and the calculation of enantiomer ratios S10

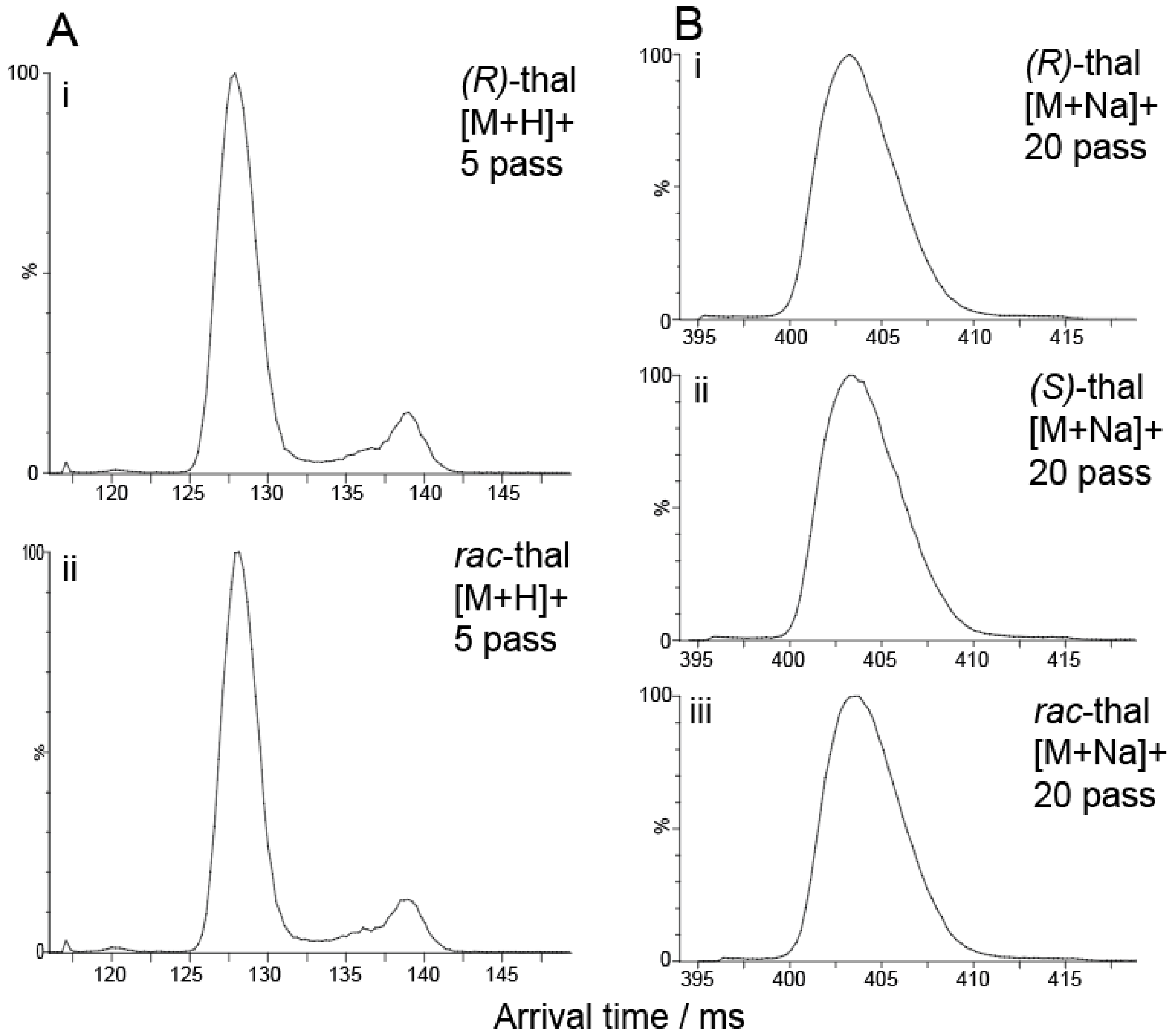

Figure S1. Multipass cyclic IMS of monomeric thal. A) Five pass cIMS experiment of the [M+H]+ of (*R*)-thal (top) and *rac*-thal (bottom). The arrival time distributions are highly similar indicating that no enantiomer separation is observed. B) Twenty pass cIMS of the [M+Na]+ ion of (*R*)-thal (i), (*S*)-thal (ii) and *rac*-thal (iii). The arrival time distributions are identical, again indicating that no enantiomer separation was observed.

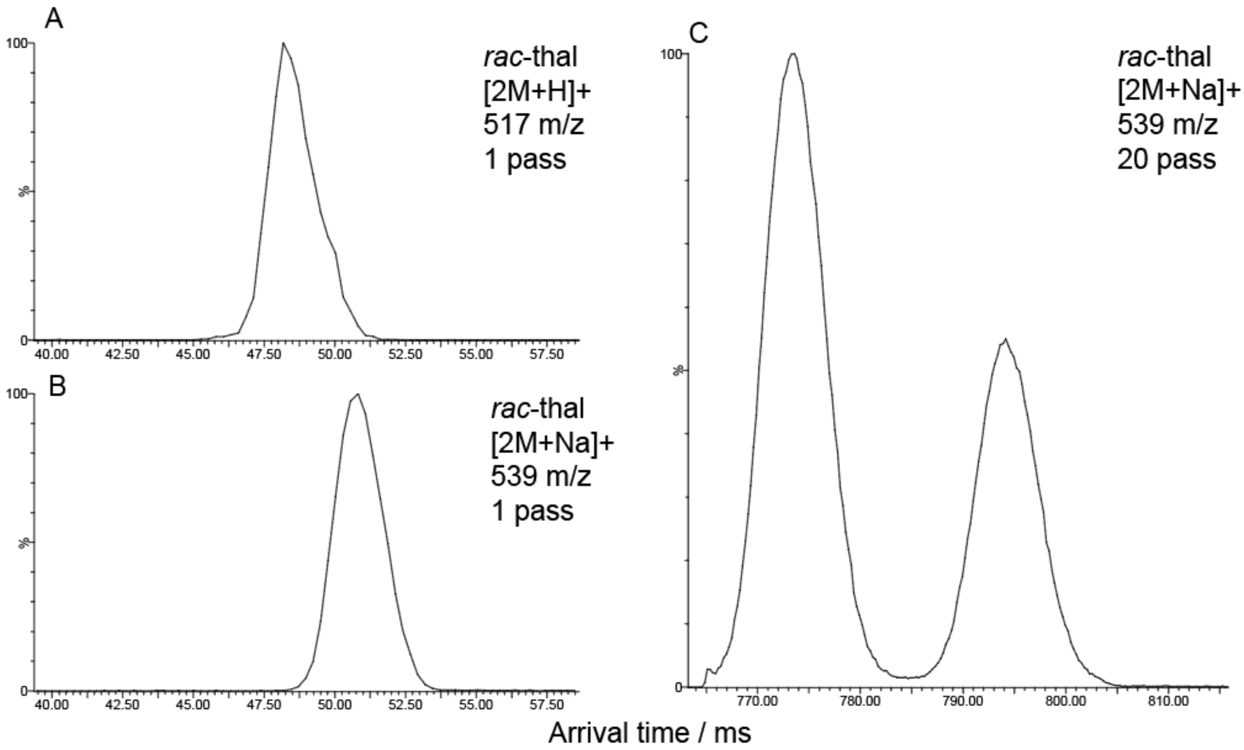

Figure S2. Single and multipass cyclic IMS of dimerc thal. A) Single pass cIMS of the [2M+H]+ of *rac*-thal showing no separation, although the ATD is asymmetric indicating two distributions The mobility resolving power is ~65 CCS/ΔCCS. B) Single pass cIMS of the [2M+Na]+ of *rac*-thal, again showing no separation (R ~ 65 CCS/ΔCCS). C) These latter ions after 20 cIMS passes showing near baseline separation (R ~ 290 CCS/ΔCCS).

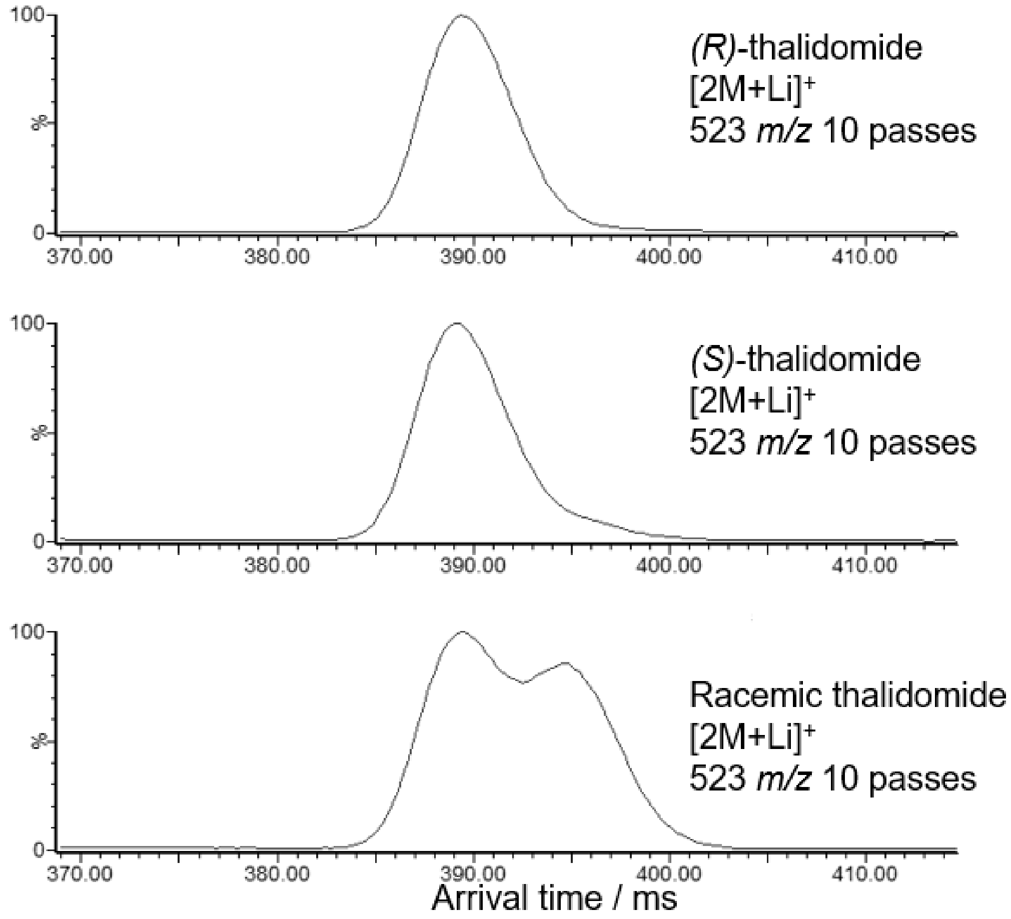

Figure S3. Ten pass cyclic ion mobility experiments of lithium adducts of thalidomide dimers at 523 *m/z*. In the same way as for the other adducts the (*R*)-thal dimer distributions appear identical to the (*S*)-thal dimer distributions. The data from *rac*-thal has two features, again in agreement with the other adducts.

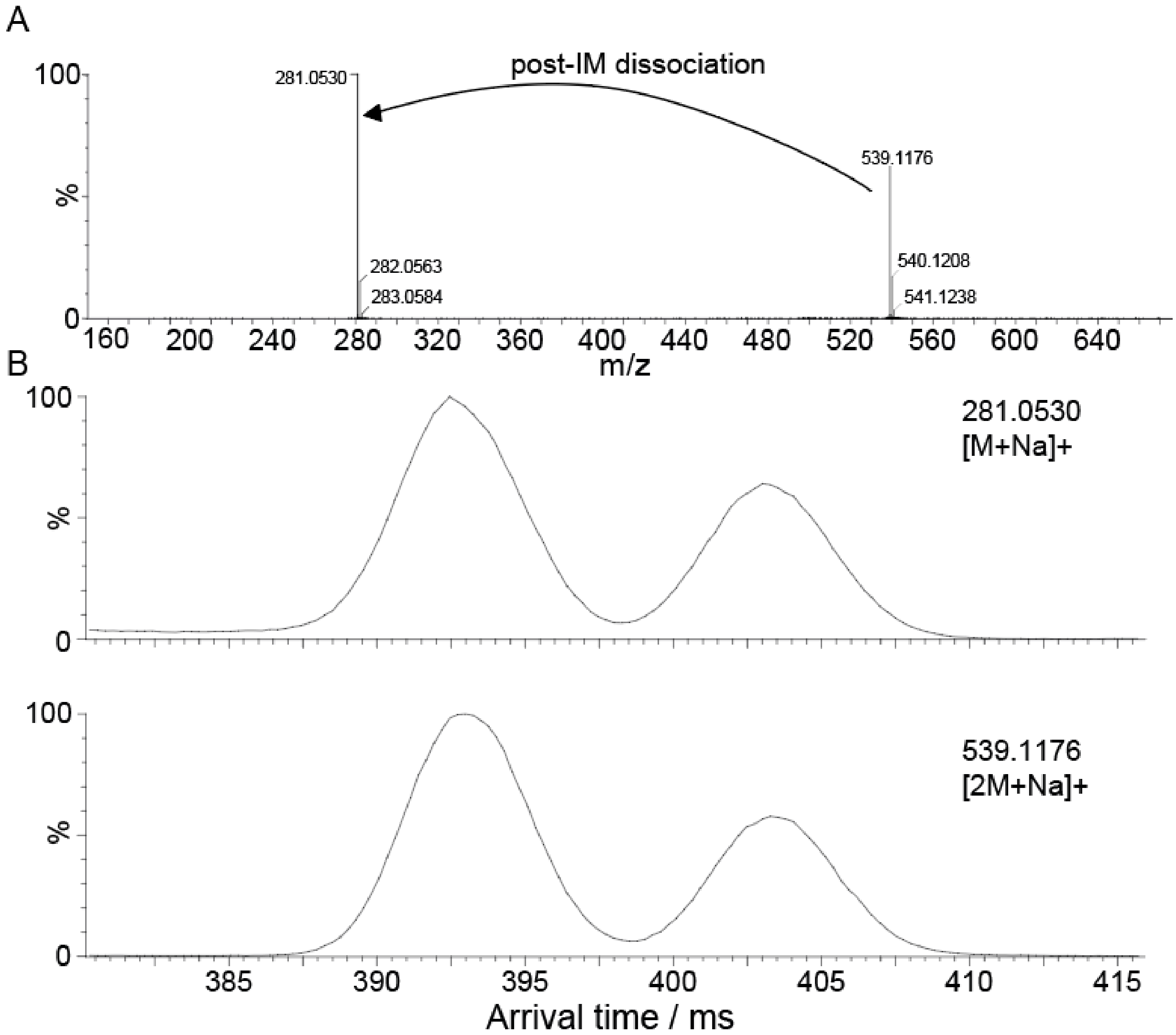

Figure S4. The pitfalls of harsh tuning on the dimer separation experiments. A) With harsh tuning post-mobility a significant amount of thal dimers dissociate into monomers. B) Extracting the mobilogram of the monomer at 281 *m/z* shows the characteristic two features of the dimers as seen for the extracted mobilogram of 539 *m/z*. Without optimized tuning the dimers may completely convert to monomers leading to misinterpretation of the ATDs as enantiomer separation. We therefore recommend milder instrument tuning to preserve the dimers as the predominant species.

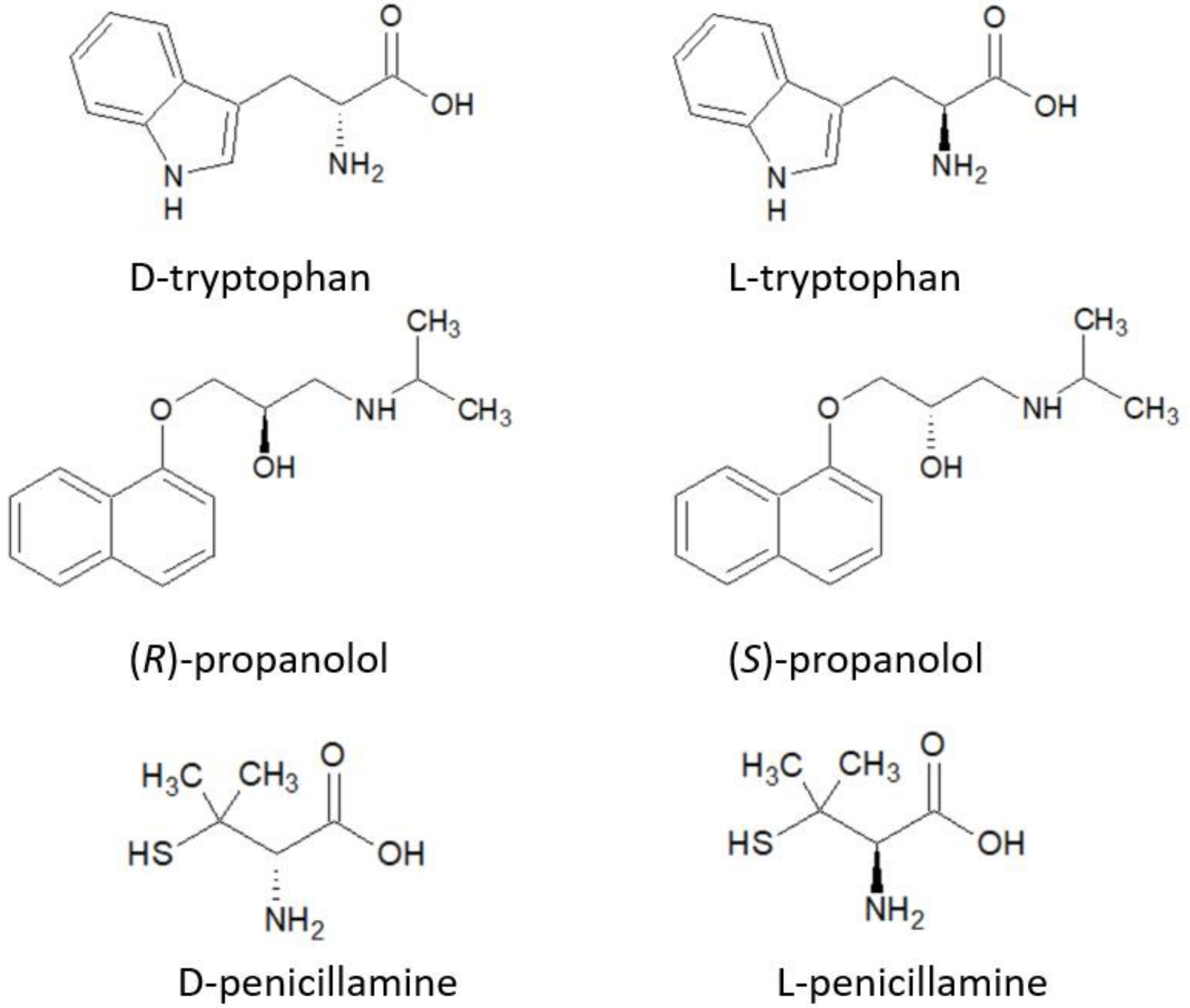

Figure S5. Structures of the chiral compounds studied in this work. From top left to bottom right; D-tryptophan, L-tryptophan, (*R*)-propanolol, (*S*)-propanolol, D-penicillamine and L-penicillamin.

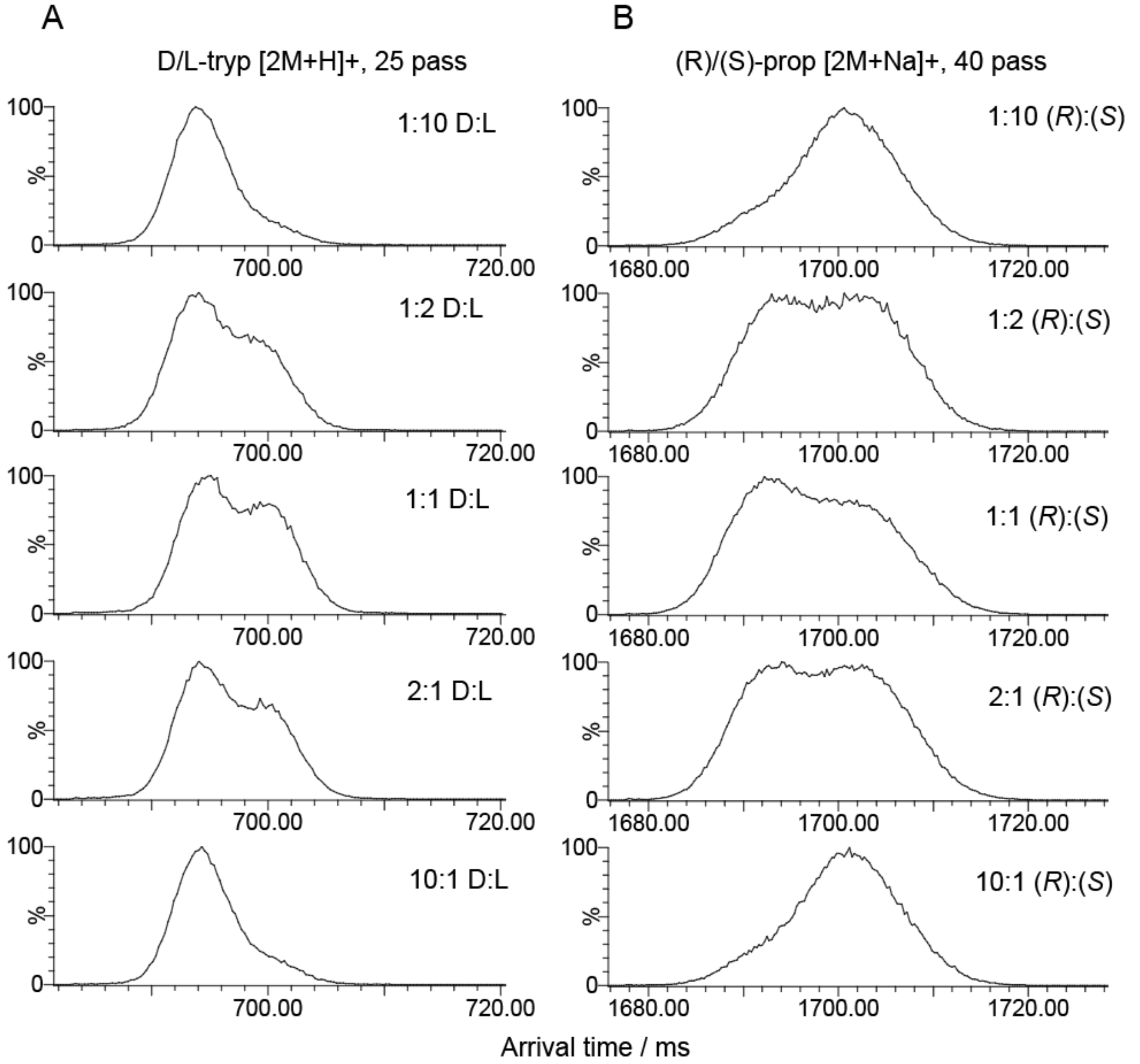

Figure S6. Arrival time distributions of the [2M+H]+ of D/L-tryp (A) and [2M+Na]+ of (R)/(S)-prop (B) at ratios of 10:1, 2:1, 1:1, 1:2 and 1:10. The relative areas of the two features vary with the ratios.

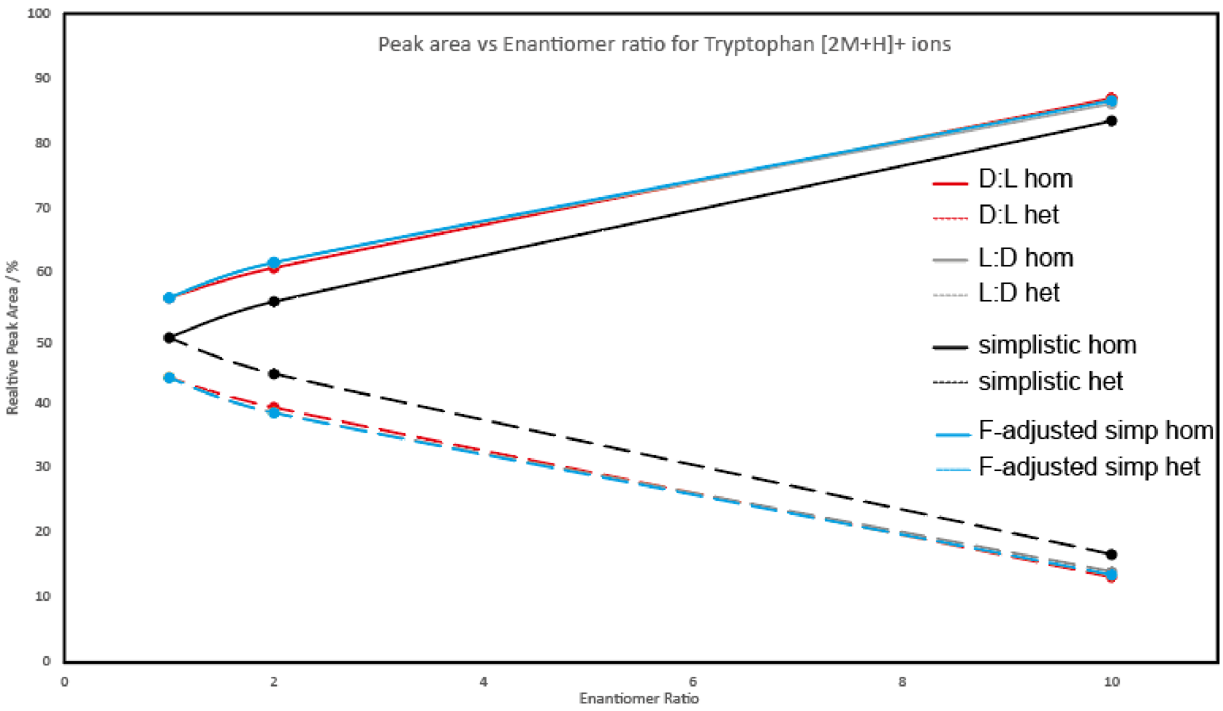

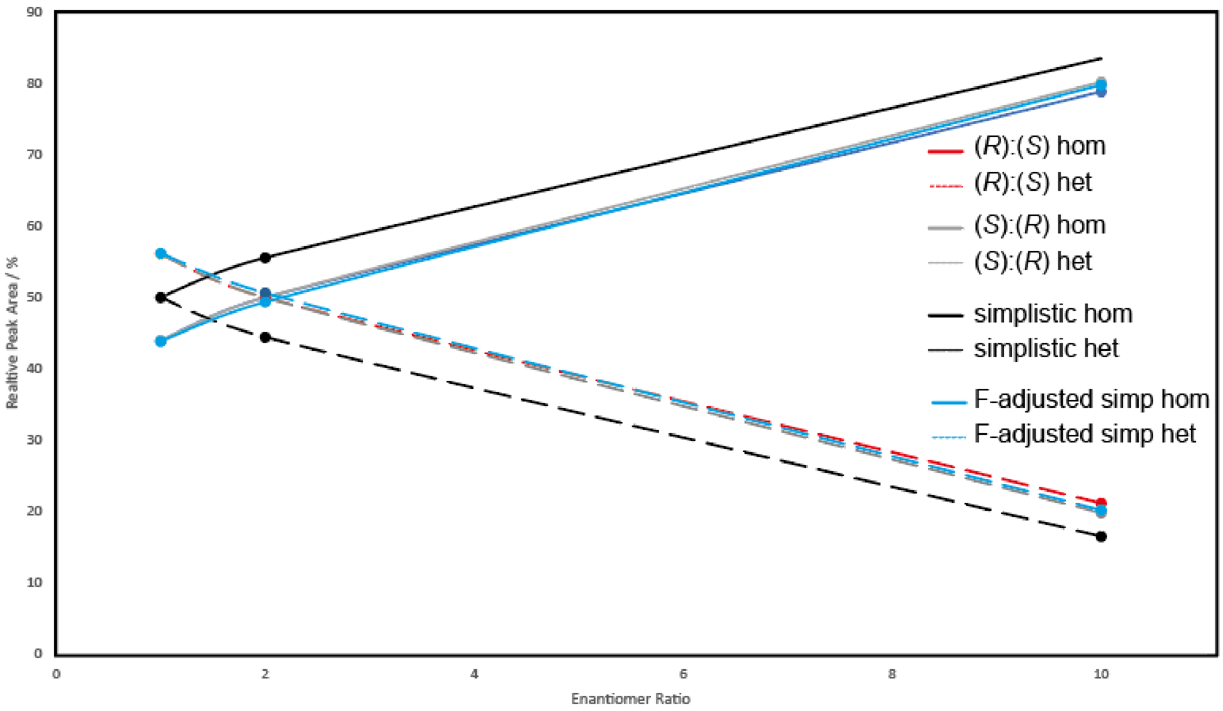

Figure S7. Plots of relative peak areas vs enantiomer ratios for D/L-tryp and (R)/(S)-prop.

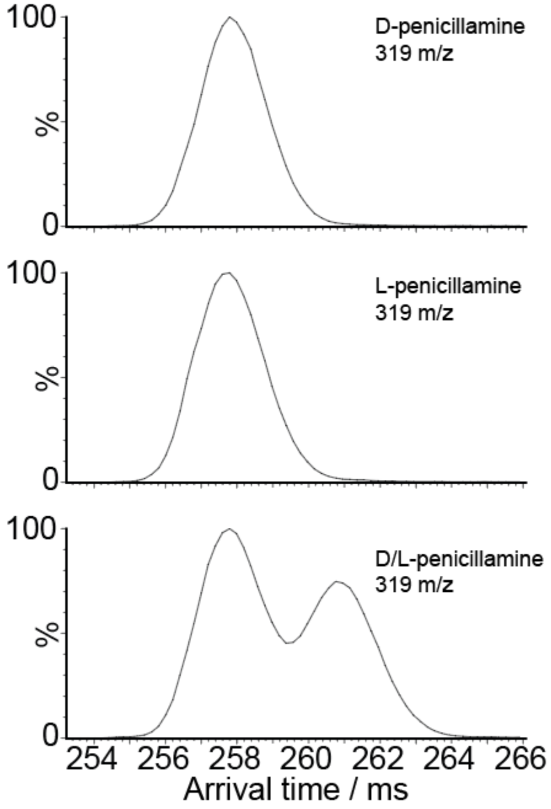

Figure S8. Ten pass cIMS of covalent dimers of D/L-penicillamine.

Supplementary Methods – Calculation of theoretical relative peak areas for homo- and heterodimeric species and the calculation of enantiomer ratios

Theoretical relative peak areas for the homodimer and heterodimer features were calculated as follows

$$I_{hom} = P_{RR} + P_{SS} \tag{1}$$

$$I_{het} = P_{RS} + P_{SR} = 2P_{RS} \tag{2}$$

Where I_{hom} and I_{het} are the relative intensities of the homodimer feature and the heterodimer feature, respectively. P_{RR}, P_{SS}, P_{RS} and P_{SR} are the probabilities of randomly forming the corresponding homodimers (*RR* and *SS*) and heterodimers (*RS* and *SR*).

And

$$P_{RR} = \left(\frac{[R]}{[R]+[S]}\right)^2 \tag{3}$$

$$P_{SS} = \left(\frac{[S]}{[R]+[S]}\right)^2 \tag{4}$$

$$P_{RS} = \left(\frac{[R]}{[R]+[S]}\right) \times \left(\frac{[S]}{[R]+[S]}\right) \tag{5}$$

$$P_{SR} = \left(\frac{[S]}{[R]+[S]}\right) \times \left(\frac{[R]}{[R]+[S]}\right) \tag{6}$$

[*R*] and [*S*] are the starting concentrations of the *R* and *S* enantiomers, respectively.

Let us consider the case where the (*R*) enantiomer is in excess and that we can describe its concentration as a relative concentration, $[R]_{rel}$, because we are concerned only with the enantiomer ratio. This consideration also makes $[S]_{rel} = 1$ (so that in a 50:1 R:S enantiomer ratio solution $[R]_{rel} = 50$ and $[S]_{rel} = 1$). Substituting equations 3-6 into 1 and 2 gives:

$$I_{hom} = \frac{[R]_{rel}^2 + 1}{(R_{rel}+1)^2} \tag{7}$$

$$I_{het} = 1 - \frac{[R]_{rel}^2 + 1}{(R_{rel} + 1)^2} \tag{8}$$

In the non-simplistic case where the homodimer and heterodimer species have different association energies we invoke the response factor, *F*:

$$I_{hom,adj} = \frac{I_{hom}}{I_{hom} + FI_{het}} \tag{9}$$

and

$$I_{het,adj} = \frac{FI_{het}}{I_{hom} + FI_{het}} \tag{10}$$

where $I_{hom,adj}$ and $I_{het,adj}$ are the response factor-adjusted relative intensities of the homodimer and heterodimer, respectively. As an example, in the case of thalidomide, $F = 0.52$. The homodimer can be considered to have a response factor but it is equal to 1 in all cases so we can ignore it.

In terms of $[R]_{rel}$ as a proxy for the enantiomer ratio (*E.R.*):

$$I_{hom,adj} = 1 - \frac{2[R]_{rel}F}{2[R]_{rel}F + [R]_{rel}^2 + 1} \tag{11}$$

$$I_{het,adj} = \frac{2[R]_{rel}F}{2[R]_{rel}F + [R]_{rel}^2 + 1} \tag{12}$$

Equations 11 and 12 are quadratic in $[R]_{\mathrm{rel}}$ and so rearranging for $[R]_{\mathrm{rel}}$ gives two roots. The roots are $[R]_{\mathrm{rel}}$ and $1/[R]_{\mathrm{rel}}$ meaning the non-decimal root should be taken. The non-decimal roots are obtained by either using $I_{hom,obs}$ or $I_{het,obs}$, (instead of the theoretical $I_{hom,adj}$ and $I_{het,adj}$) which are the observed relative peak areas of the homodimer and heterodimer measured in the experiment, respectively, and *F*, the heterodimer response factor. Using $I_{hom,obs}$:

$$[R]_{rel} = \frac{-(\sqrt{I_{hom,obs}^2 F^2 - I_{hom,obs}^2 + 2I_{hom,obs} - 1} + I_{hom,obs}F)}{I_{hom,obs} - 1} = E.R. \tag{13}$$

Similarly, using $I_{het,obs}$:

$$[R]_{rel} = \frac{\sqrt{(I_{het,obs}^2 - 2I_{het,obs} + 1)F^2 - I_{het,obs}^2} + (1 - I_{het,obs})F}{I_{het,obs}} = E.R. \tag{14}$$

Remember that $[R]_{rel}$ in equations 7-14 assume the *R* enantiomer is in excess. If the *S* enantiomer is in excess all instances of $[R]_{rel}$ could be substituted with $[S]_{rel}$. Equations 13 and 14 are equivalent equation 2 in the main text.
